# Patient-specific mutation of contact site protein Tomm70 causes neurodegeneration

**DOI:** 10.1242/dmm.052029

**Published:** 2025-04-28

**Authors:** Vranda Garg, Wiebke Möbius, Ralf Heinrich, Torben Ruhwedel, Roshan Priyarangana Perera, Patricia Scholz, Till Ischebeck, Gabriela Salinas, Christian Dullin, Martin C. Göpfert, Jacob Engelmann, Roland Dosch, Bart R. H. Geurten

**Affiliations:** ^1^ Department of Cellular Neurobiology, Georg-August-University Göttingen, 37077 Göttingen, Germany; ^2^ Department of Neurogenetics, Max Planck Institute for Multidisciplinary Sciences, 37075 Göttingen, Germany; ^3^ Department of Developmental Biochemistry, Georg-August-University Göttingen, 37077 Göttingen, Germany; ^4^ Department of Plant Biochemistry, Albrecht-von-Haller-Institute for Plant Sciences and Gottingen Center for Molecular Biosciences (GZMB) Georg-August-University Göttingen, 37077 Göttingen, Germany; ^5^ Institute of Human Genetics, University Medical Center, Göttingen Georg-August-University Göttingen, 37073 Göttingen, Germany; ^6^ Department of Diagnostic and Interventional Radiology, University Medical Center, Göttingen, Georg-August-University Göttingen, 37075 Göttingen, Germany; ^7^ Faculty of Biology, Bielefeld University 33615 Bielefeld, Germany; ^8^ Department of Zoology, University of Otago 39054 Dunedin, New Zealand

**Keywords:** Tomm70, Zebrafish, Neurodegeneration, Endoplasmic reticulum-mitochondria contact site

## Abstract

TOMM70 is a receptor at the contact site between mitochondria and the endoplasmic reticulum, and *TOMM70* has been identified as a risk gene for hereditary spastic paraplegia. Furthermore, *de novo* missense variants of *TOMM70* have been identified to cause neurological impairments in two unrelated patients. Here, we show that mutant zebrafish *ruehrei^p25ca^* also harbour a missense mutation in *tomm70*, affecting the same conserved isoleucine residue as in one of the human patients. Using this model, we demonstrate how loss of Tomm70 function leads to impairment. At the molecular level, the mutation affected the interaction of Tomm70 with the endoplasmic reticulum protein Lam6, a known sterol transporter. At the neuronal level, the mutation impaired mitochondrial transport to the axons and dendrites, leading to demyelination of large calibre axons in the spinal cord. These neurodegenerative defects in zebrafish were associated with reduced endurance and swimming efficiency, and alterations in the C-start escape response, which correlated with decreased spiking in giant Mauthner neurons. Thus, in zebrafish, a mutation in the endoplasmic reticulum-mitochondria contact site protein Tomm70 recreates some of the neurodegenerative phenotypes characteristic of hereditary spastic paraplegia.

## Teaser

**Summary:** A mutation in the mitochondrial receptor protein Tomm70 disrupts neuronal and molecular processes in zebrafish, providing critical insights into the neurodegenerative mechanisms underlying hereditary spastic paraplegia.

## INTRODUCTION

Translocase of outer mitochondrial membrane 70 (Tomm70) is a 70 kDa mitochondrial import receptor protein composed of 26 α helices, the majority of which contribute to the formation of tetra-trico-peptide repeat (TPR) motifs. These TPR motifs mediate the interaction of Tomm70 with other proteins (Kreimendahl and Rassow, 2020). Although the role of Tomm70 in facilitating the uptake of proteins into the mitochondria is well established, recent studies have shown that Tomm70 is also a key component of the endoplasmic reticulum (ER)-mitochondria contact site (MCS) (Filadi et al., 2018), where it binds with an ER-localised sterol transporter protein Lam6 (also known as Ltc1) (Murley et al., 2015). ER-MCSs are known to regulate a number of key cellular processes, including lipid metabolism, calcium homeostasis, mitochondrial functions, ER stress and autophagy, and the defects in contact site proteins are associated with neurodegenerative disorders (Wilson and Metzakopian, 2021). *TOMM70* has been reported to be a potential candidate gene for hereditary spastic paraplegia (HSP) (Novarino et al., 2014). HSPs are a complex group of neurodegenerative disorders, characterised by progressive lower-limb spasticity and weakness, that result from the degeneration of corticospinal motor neurons (Blackstone, 2012, 2018; Fink, 2006, 2014; Finsterer et al., 2012; Harding, 1984, 1993; Klebe et al., 2015; Tesson et al., 2015)_._

Although the pathophysiology of HSP is well described, (Blackstone, 2012, 2018; Elsayed et al., 2021; Fink, 2006, 2014; Finsterer et al., 2012; Giudice et al., 2014; Harding, 1993, 1984; Klebe et al., 2015; Parodi et al., 2018; Tesson et al., 2015), little is known about its aetiology. A maternal effect mutant screen in zebrafish (Dosch et al., 2004) generated several lines characterised by opaque eggs. Here, we show that one of these, *ruehrei^p25ca^*, possess a single base change that changes the conserved isoleucine at position 525 of Tomm70 to a threonine (Tomm70^Ile525Thr^). Mutation of the corresponding isoleucine to phenylalanine (TOMM70^Ile554Phe^) was reported to lead to dystonia, hypotonia, hyper-reflexia, ataxia, dysarthria, tremor, ptosis and white-matter abnormality in a human patient (Dutta et al., 2020). Here, we used this zebrafish model to examine the effects of this missense mutation at the molecular, neuronal, physiological and behavioural levels, providing insights into its potential role in neurodegenerative processes.

Using bimolecular fluorescence complementation (BiFC) assay, we found that, in zebrafish, the mutation leads to a partial impairment in the interaction between Tomm70 and Lam6 at the ER-MCS. Reduced interaction with this sterol transporter was associated with a lowered level of cholesterol in the opaque eggs produced by the *tomm70* (also known as *tomm70a*) mutant zebrafish. Primary brain neuronal culture studies revealed that the non-synonymous mutation also affected the transport of mitochondria into axons and dendrites. Using high-pressure freezing and electron microscopy, we demonstrated that the mutation affects the integrity of the myelin sheath and that *tomm70* mutants show signs of demyelination of the large calibre axons in the spinal cord. These physiological changes were accompanied by locomotion defects. The *tomm70* mutants could not maintain continuous swimming for extended periods, owing to their reduced endurance and diminished propulsion efficiency. The *tomm70* mutants also showed defective axial bending during the C-start escape response. This indicated that Mauthner neurons, giant rapid-firing neurons that drive this rapid escape response, were also affected by the mutation in the *tomm70* gene.

Hence, in our zebrafish model, we show how a missense mutation alters the functioning of an ER-MCS protein, leading to demyelination of the spinal cord neuron and subsequent functional degeneration. This provides evidence that mutations affecting the function of *tomm70* causes neurodegenerative symptoms.

## RESULTS

### Missense mutation at the highly conserved Ile525 reduces the amount of Tomm70 protein

The zebrafish mutant *ruehrei^p25ca^*, identified in a maternal-effect mutant screen, shows defective stage IV oocyte maturation (Dosch et al., 2004). The mutant produces opaque eggs that fail to undergo cell division, suggesting a critical role for the affected gene in oogenesis. We used RNA sequencing and analysis to show that the *ruehrei^p25ca^* mutants carry a single point mutation in the *tomm70* gene. This mutation results in a base change from thymine to cytosine, leading to an amino acid substitution from isoleucine to threonine at position 525 in the C-terminus of the protein Tomm70 (Fig. S1A,B). The amino acid isoleucine affected by the Tomm70^Ile525Thr^ mutation is highly conserved across vertebrate orthologues of *Danio rerio* Tomm70, including human TOMM70 (Fig. S1C). In human TOMM70, the equivalent isoleucine resides at amino acid position 554, and its substitution by phenylalanine, TOMM70^Ile554Phe^, causes neurological defects in a patient, including involuntary muscle contraction, decreased muscle tone, abnormal posture, spastic tendencies, white-matter abnormalities and ataxia (Dutta et al., 2020). Using western blot analysis, we found that the *Danio rerio* Tomm70^Ile525Thr^ mutation reduced the abundance of the Tomm70 protein in the brain of adult female and male *tomm70* heterozygous mutants. The reduction in protein abundance intensified in homozygous mutants to ∼50% (Fig. S1D-G).

To explore whether the *Danio rerio* Tomm70^Ile525Thr^ mutation compromises Tomm70 protein structure, we compared the predicted structures of wild-type and mutant protein using the PyMol application and the template modelling (TM)-align server of Iterative Threading ASSEmbly Refinement (I-TASSER) (Zhang and Skolnick, 2005). A value of 0.233 for the root-mean-square deviation of atomic positions and 0.83586 for TM score, a measure of similarity between two protein structures, indicated that the mutation has no effect on the structural conformation of the Tomm70 protein (Fig. S2A-C).

### Partial loss of interaction of Tomm70 with Lam6 at the ER-MCS

Tomm70 is an outer mitochondrial membrane protein, known to interact with Tomm40, another mitochondrial protein, and with Lam6, an ER protein responsible for sterol transport (Murley et al., 2015; Tang et al., 2019). To assess whether the mutation might affect these interactions, we used a BiFC assay, in which two proteins are tagged with unfolded complementary N- and C-terminal fragments of a fluorescent protein reporter (Kerppola, 2006, 2008). We used N-terminal and C-terminal split fragments of the yellow fluorescent protein Venus as the reporter, termed ‘VN’ and ‘VC’, respectively (Fig. S3A). *Danio rerio* Tomm70 was tagged with VN and yeast Lam6 with VC. We cloned the *LAM6* gene from yeast genomic DNA because, for *Danio rerio*, there are eight different Lam6 homologues with different transcripts. Four different combinations were tested: Tomm70-VN+VC-Lam6, Tomm70-VN+Lam6-VC, VN-Tomm70+VC-Lam6 and VN-Tomm70+Lam6-VC. Of these four different combinations, Tomm70-VN+VC-Lam6 was the only combination that generated fluorescence (Fig. S3C and Fig. S4A-F). We repeated the experiment using all four possible combinations with mutant Tomm70^Ile525Thr^, and, in this case as well, fluorescence was observed only for Tomm70^Ile525Thr^-VN+VC-Lam6 (Fig. S3C and Fig. S4A-F). Quantification revealed a significant decrease in the percentage of fluorescent embryos for the mutant Tomm70 combination compared to the wild-type combination (Fig. S3D). The reduced fluorescence indicates that the Tomm70^Ile525Thr^ mutation likely increases the spatial separation between Lam6 and Tomm70.

Next, we investigated whether the Tomm70^Ile525Thr^ mutation affects the interaction with Lam6 only or also that with Tomm40. We tagged Tomm40 with VC and tested all four possible combinations. In this control experiment, strong fluorescence signals were observed when VN was attached to the C-terminus of Tomm70 with both Tomm40-VC and VC-Tomm40 (Fig. S3E and Fig. S5A). On quantification, we found no change in the percentage of fluorescent-positive embryos between wild type and mutant for the two combinations (Fig. S3F and Fig. S5B). The attachment of VN to the N-terminus of Tomm70 yielded only minimal fluorescence, rendering any fluorescence differences between mutant and wild type negligible, given the considerably lower absolute fluorescence levels observed (Fig. S5C-F).

Yeast Lam6 reportedly acts as a transporter of sterols at the ER-MCS (Murley et al., 2015). Because the Tomm70^Ile525Thr^ mutation impairs the interaction with Lam6, we wondered whether it might affect sterol levels, such as those of cholesterol. Using gas chromatography-mass spectrometry (GC-MS), we determined cholesterol levels in the eggs of wild-type fish and *tomm70* mutants. Cholesterol levels were significantly lowered in the mutant opaque eggs (Fig. S3H).

### Absence of Tomm70 from axons in mutant fish

**Fig. 1.**
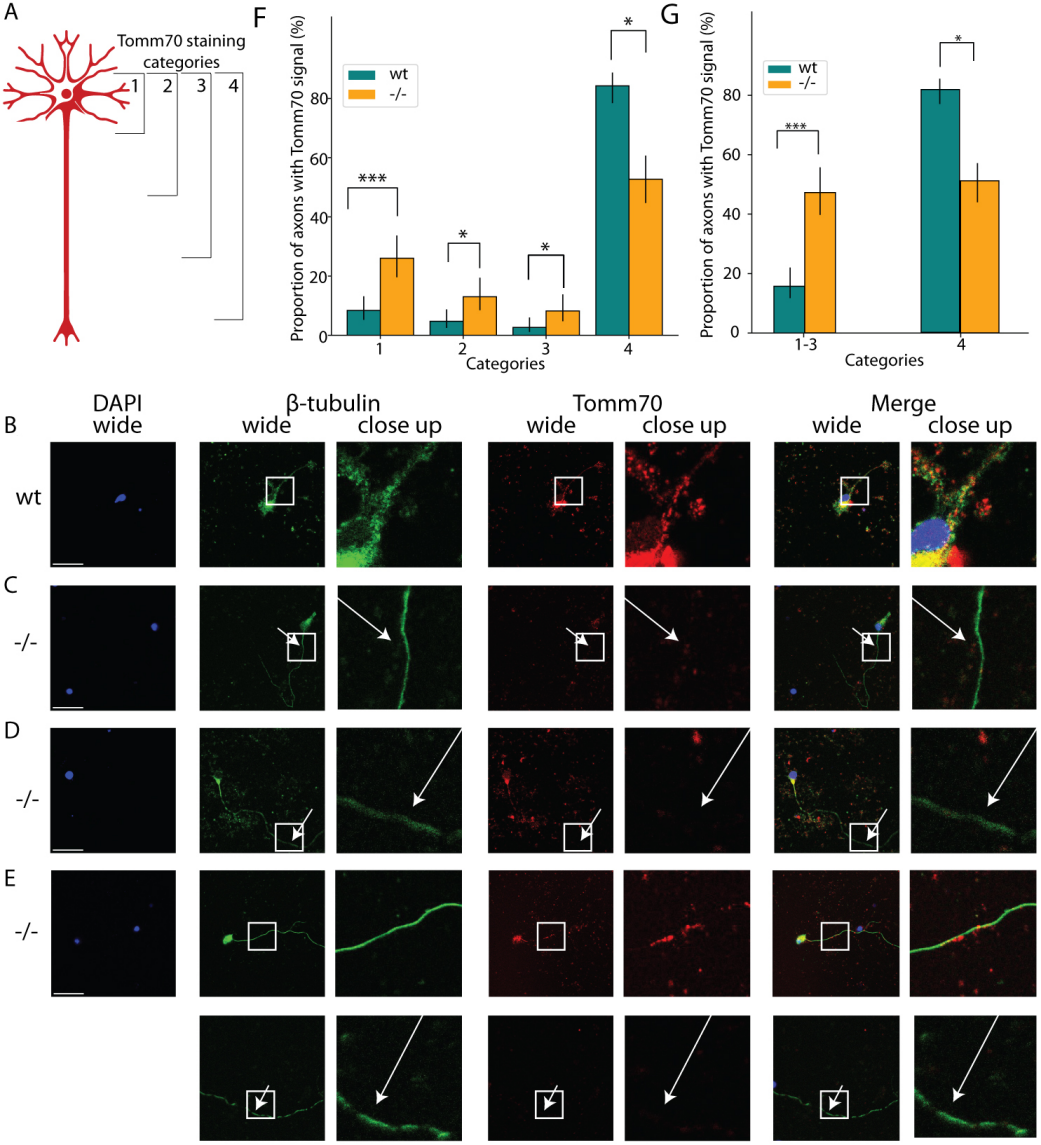
**Mutation leads to the absence of Tomm70 from axons.** The cultured brain neurons of wild-type (wt) and *tomm70* mutant female fish were stained with DAPI (representing nucleus; blue), anti-β-Tubulin antibody (a neuronal marker) (green) and anti-Tomm70 antibody (red). The brightness of images was corrected using ImageJ. (A) The diagram represents four different categories of staining observed during imaging and quantification of the wt and *tomm70* mutant brain cultured neurons stained with anti-Tomm70 antibody. Categories 1, 2, 3 and 4 depict the location of the signal for Tomm70 in the neurons. (B-E) Representative images of each category of Tomm70 staining in neurons. Scale bars: 30 µm. (B) wt fish showing signal for Tomm70 both in the soma and axon (category 4). (C) *tomm70* fish showing signal for Tomm70 only in the soma (category 1). (D) *tomm70* fish showing signal for Tomm70 in the soma and in the initial part of the axon (category 2). (E) *tomm70* fish showing signal for Tomm70 in the soma and half way along the axon (category 3). The white-line box in the wide-field column highlights the specific region magnified in the adjacent close-up column. White arrows in the mutants for both β-Tubulin and Tomm70 staining indicate identical locations, underscoring the absence of Tomm70 in these areas. (F) Quantification representing the percentage of neuronal staining in four different categories for wt and mutants. More than 80% of the neuronal staining in wt was classified as category 4. Conversely, in the mutants, there was a significantly higher percentage of neuronal staining in categories 1, 2 and 3, and a significantly lower percentage of neuronal staining in category 4. (G) Quantification of the percentage of neuronal staining in categories 1-3 together, and for category 4, in wt and mutant fish. There is a significant increase in the percentage of neuronal staining in categories 1-3 in mutants compared to wt. Number of fish (*N*)=9 (wt) and 7 (−/−); total number of neurons counted (*n*)=191 (wt) and 146 (−/−). Error bar represents 95% c.i. Statistical significance was tested using Fisher's permutation test. *P<0.05 and ***P<0.001. ‘−/−’ in this and other figures refers to *Danio rerio* Tomm70^Ile525Thr^ mutants, which possess a missense mutation, not a null mutation.

As our study revealed a partial loss in the interaction of Tomm70 with Lam6 and a potential consequence on sterol levels, we investigated the effects of this mutation on the central nervous system. We used primary neuronal culture and immunocytochemistry to examine the presence of Tomm70 in the brain neurons of the mutant fish. Fig. 1A illustrates the different types of neuronal staining with the anti-Tomm70 antibody. In order to quantify the staining, we devised four categories: category 1 represents the most severe phenotype, with antibody signals for Tomm70 only present in the soma and absent from the axon; category 2 includes neuronal staining in which the Tomm70 signal was observed in the soma and the initial part of the axon; in category 3, Tomm70 signal was present in the soma and halfway along the axon; and category 4 represents the neuronal staining with signal for Tomm70 in the soma and throughout the axon. Fig. 1B represents typical neuronal staining found in female wild-type fish, which predominantly belonged to category 4. Fig. 1C-E display representative images of neuronal staining predominantly observed in mutant female fish brains, corresponding with categories 1, 2 and 3, respectively. More than 80% of the neuronal staining in the brain of wild-type female fish belonged to category 4, whereas there was a significant decrease in the percentage of staining in this category in the mutant female fish. Conversely, there was a significant increase in the percentage of staining in categories 1, 2 and 3 in mutant female fish compared to in the wild-type female fish (Fig. 1F). Compared to that in wild types, there was still a significant increase in the percentage of neuronal staining in mutants when the data for categories 1, 2 and 3 were pooled (Fig. 1G). Although male fish showed no significant changes in categories 2-4, the most severe phenotype, category 1, was significantly increased in male mutants compared to male wild types (Fig. S7F). All trends were similar to those of the female data, and pooled categories were significantly different (Fig. S7G). These findings revealed absence of Tomm70 from the axons in a length-dependent manner.

### Tomm70^Ile525Thr^ mutation impairs axonal and dendritic mitochondrial transport

**Fig. 2.**
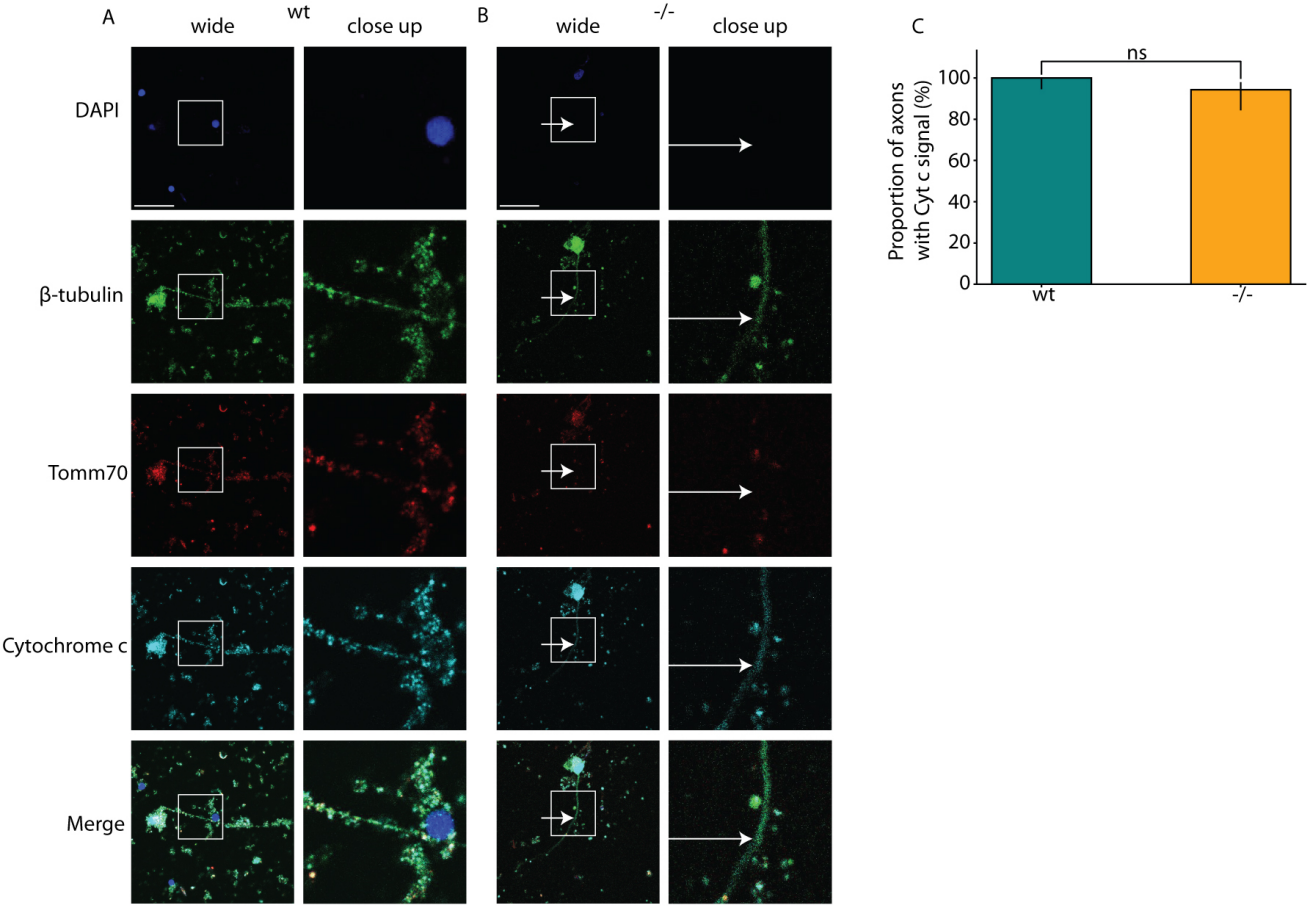
**Mutation impacts the transport of mitochondria to the axons.** The cultured brain neurons of wt and *tomm70* mutant female fish were stained with DAPI (representing nucleus; blue), anti-β-Tubulin antibody (a neuronal marker; green), anti-Tomm70 antibody (red) and anti-Cytochrome c antibody (a conserved mitochondrial marker; cyan). The brightness of images was corrected using ImageJ. (A,B) Representative pictures of neuronal staining of wt (A) and mutant (B) fish with anti-β-Tubulin, anti-Tomm70 and anti-Cytochrome c antibodies. The white-line box in the wide-field column highlights the specific region magnified in the adjacent close-up column. White arrows in the mutants for β-Tubulin, Tomm70 and Cytochrome c staining indicate identical locations, underscoring the absence of Tomm70 and presence of Cytochrome c in these areas. Scale bars: 30 µm. (C) Quantification of the percentage of neuronal staining showing signal for Cytochrome c in the axons in wt and *tomm70* mutants. Quantifications for Tomm70 signals are shown in Fig. 1G. *N*=4 (wt) and 3 (−/−); *n*=66 (wt) and 53 (−/−). Error bar represents 95% c.i. Statistical significance was tested using Fisher's permutation test. ns, non-significant.

**Fig. 3.**
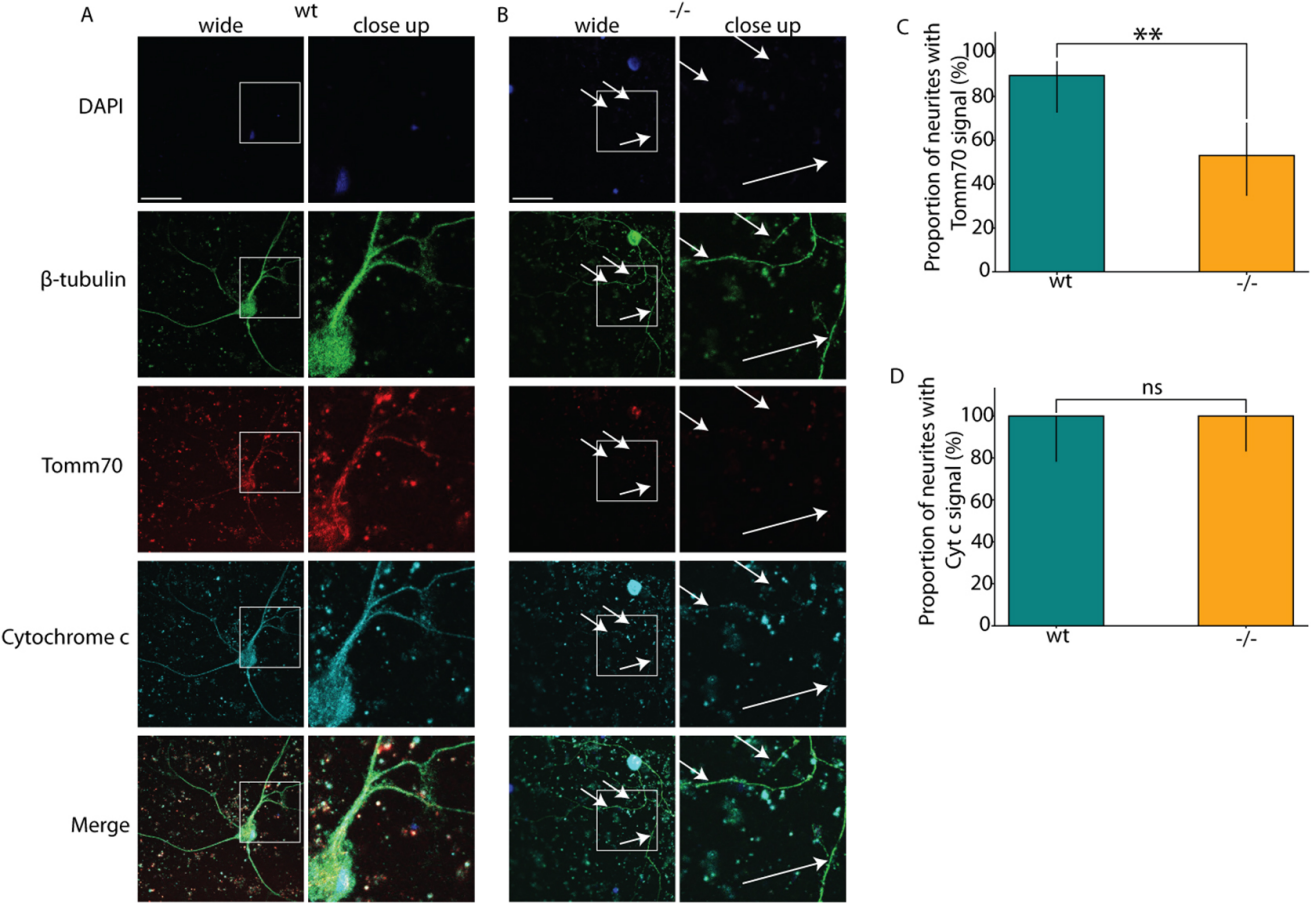
**Mutation influences the transport of mitochondria to the dendrites.** The cultured brain neurons of wt and *tomm70* mutant female fish were stained with DAPI (representing nucleus; blue), anti-β-Tubulin antibody (a neuronal marker; green), anti-Tomm70 antibody (red) and anti-Cytochrome c antibody (a conserved mitochondrial marker; cyan). The brightness of images was corrected using ImageJ. (A,B) Representative pictures of multi-polar neuronal staining of wt (A) and mutant (B) fish with anti-β-Tubulin, anti-Tomm70 and anti-Cytochrome c antibodies. The white-line box in the wide field column highlights the specific region magnified in the adjacent close-up column. White arrows in the mutants for β-Tubulin, Tomm70 and Cytochrome c staining indicate identical locations, underscoring the absence of Tomm70 and presence of Cytochrome c in all the neurites. Scale bars: 30 µm. (C) Quantification of the percentage of neuronal staining showing a signal for Tomm70 in neurites in wt and mutants. (D) Quantification of the percentage of neuronal staining showing a signal for Cytochrome c in neurites in wt and mutants. *N*=9 (wt) and 7 (−/−) for Tomm70, and *N*=4 (wt) and 3 (−/−) for Cytochrome c; *n*=29 (wt) and 32 (−/−) for Tomm70, and *n*=15 (wt) and 20 (−/−) for Cytochrome c. Error bar represents 95% c.i. Statistical significance was tested using Fisher's permutation test. **P<0.01; ns, non-significant.

Mitochondria with mutated Tomm70^Ile525Thr^ protein could not reach axon terminals (Fig. 2A,B), showing that the mutation blocks transport into axons. Mitochondria without Tomm70 protein moved freely (Fig. 2C). Similar disruptions were observed in dendrites (Fig. 3A-D) of multipolar neurons. The male mutants exhibited identical effects to those seen in female mutants (Fig. S8A-C and Fig. S9A-D).

### Demyelination in the large calibre axons of *tomm70* mutants

**Fig. 4.**
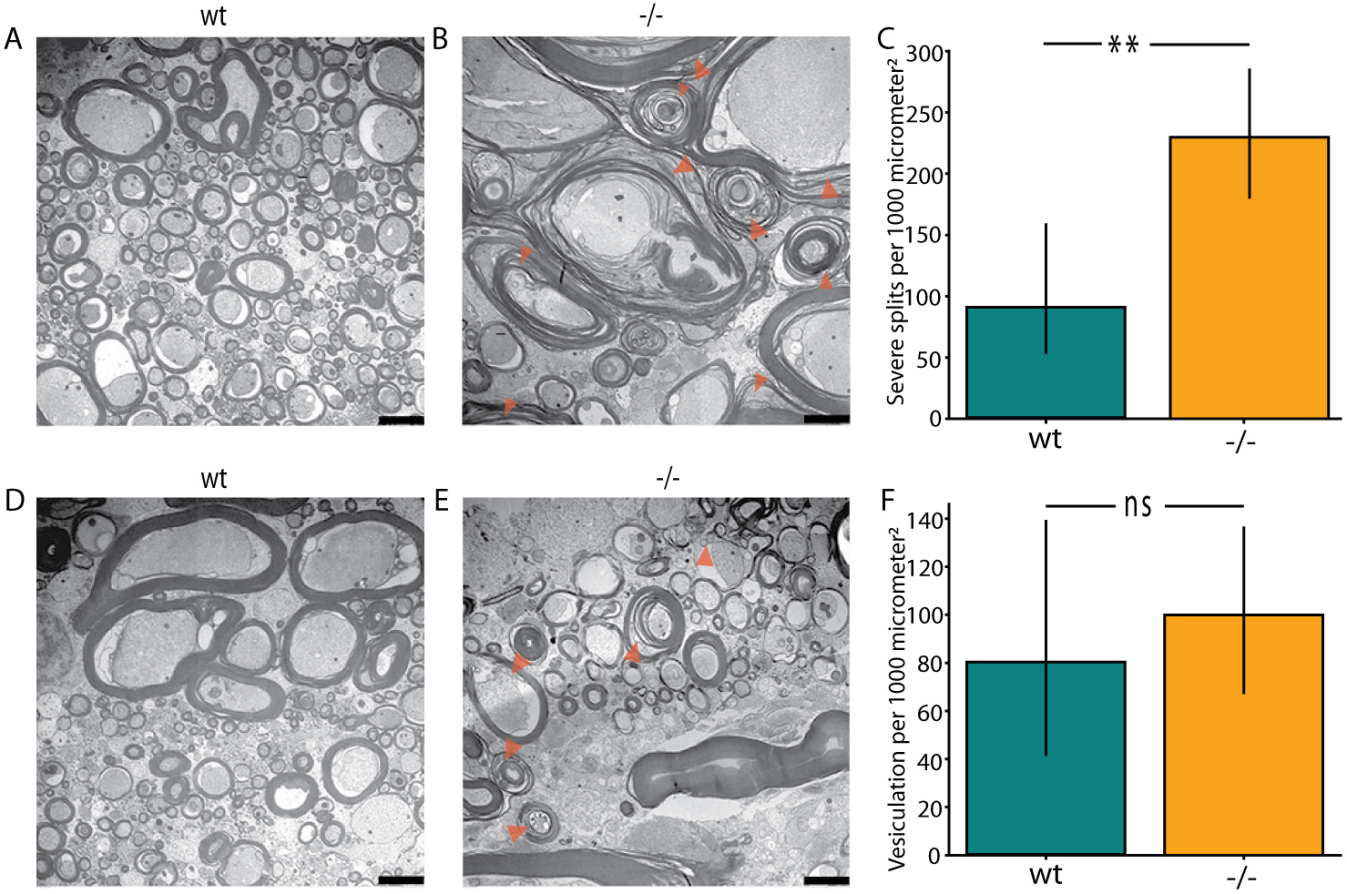
**Myelin pathology in the large calibre axons of the spinal cord of *tomm70* mutants.** (A) Representative electron microscopy picture showing large calibre axons in the cranial part of the spinal cord with intact myelin in wt fish. (B) Orange arrowheads mark points of severe splitting of myelin sheath in the large calibre axons of the cranial part of the spinal cord in homozygous mutants. (C) Quantification of severe split events of myelin per 1000 µm^2^ area in wt and *tomm70* mutant fish. There is a significant increase in the number of severe splitting cases in the cranial part of the spinal cord in mutants compared to wt. (D) Another electron micrograph showing intact myelin in the large calibre axons of the cranial part of the spinal cord in wt fish. (E) Orange arrowheads mark points of vesiculation of the myelin sheath surrounding the large calibre axons of the cranial part of the spinal cord in *tomm70* mutant fish. (F) Quantification of vesiculation events of the myelin sheath per 1000 µm^2^ area in wt and mutant fish. Although there is a slight increase in the number of cases of vesiculation in mutants in the large calibre axons of the cranial part of spinal cord, it is not changed significantly compared to that in wt. *N*=2 (wt) and 5 (−/−). Error bar represents 95% c.i. Statistical significance was tested using Fisher's permutation test. **P<0.01; ns, non-significant. Scale bars: 2500 nm.

To investigate whether the absence of Tomm70 from the mitochondria affects the structure of myelin in the large calibre axons of the spinal cord, we used electron microscopy. Two characteristic pathological features in myelin, splitting and vesiculation, were observed in the large calibre axons in the cranial portion of the spinal cord of mutants. Representative pictures of intact myelin in the cranial region of the spinal cord of wild-type fish are shown in Fig. 4A,D. Fig. 4B,E illustrate splitting and vesiculation of the myelin sheath in the cranial region of the spinal cord in mutant zebrafish. Although zebrafish myelin is inherently less compact than mammalian myelin (Siems et al., 2021), and artefacts can affect the quality of electron micrographs, quantification showed a significant increase in splitting events (Fig. 4C) and a slight increase in vesiculation of the myelin sheath in mutants compared to wild types (Fig. 4F). Although breaks in the myelin sheath can occur due to artefacts, we observed a significant increase in break events in the caudal region of the spinal cord in mutants compared to wild types (Fig. S10A-C).

### Locomotion defects in *tomm70* mutants

**Fig. 5.**
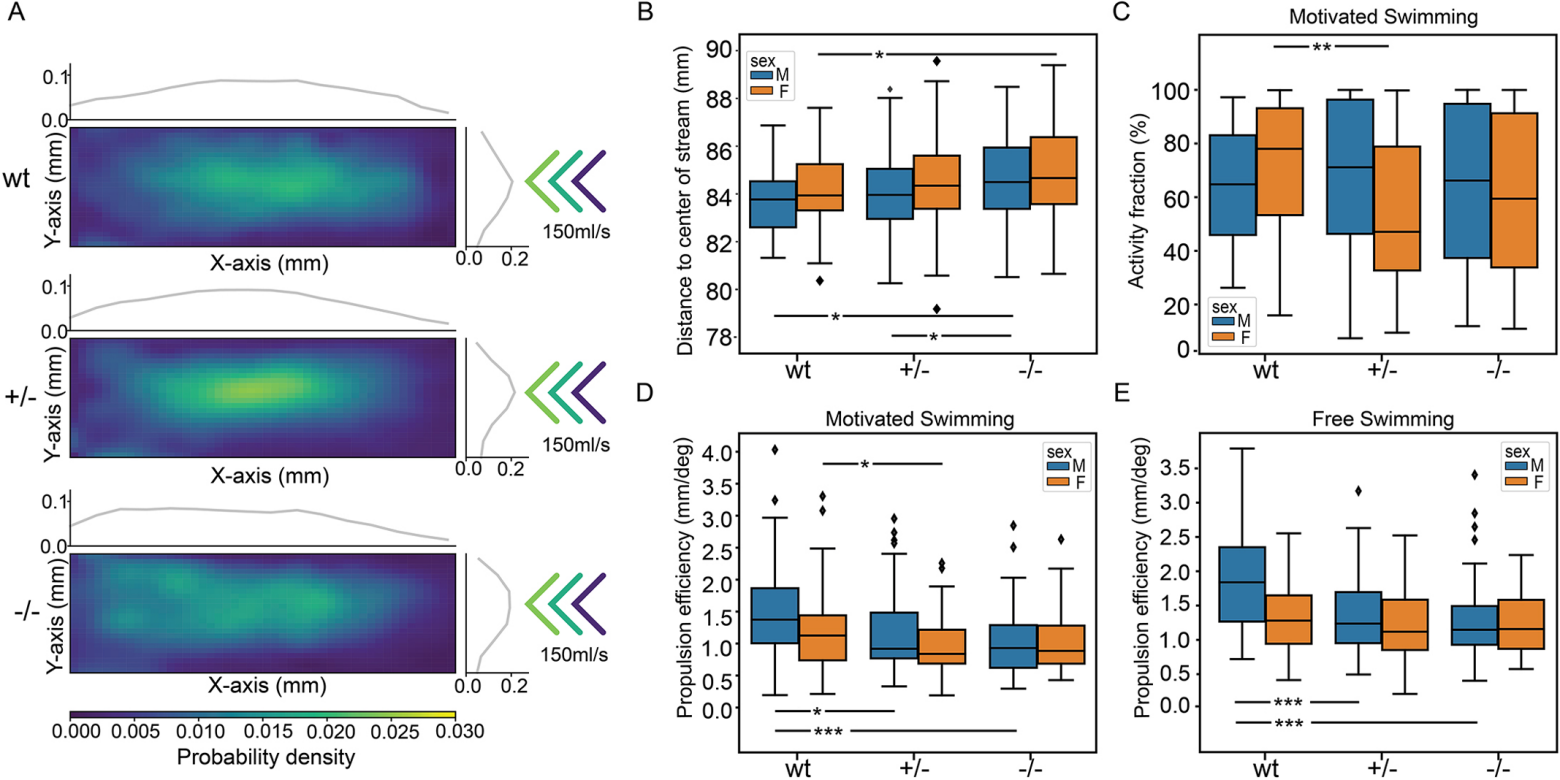
**Locomotion defects in *tomm70* mutant fish.** (A) Two-dimensional heat map with marginal histogram showing all possible locations of female fish in the setup. Blue represents low location probability; yellow represents high location probability. Although wt and heterozygous mutant female fish remain in the centre of the stream, the homozygous female mutants avoid it and reside at a longer distance from the centre of the stream. (B) Box plot of mean distance of fish to the centre of stream, propulsion efficiency and activity fraction. The black line represents the median of all individuals, the box displays the upper and lower quartiles, the whiskers denote 1.5 times the interquartile distance, and the diamonds mark outliers. There is a significant increase in the mean distance of homozygous mutant male and female fish from the centre of stream compared to that for their wt counterparts. (C) Activity is defined as the percentage of video frames in which swimming velocity exceeded 0.025 m/s. Decrease in the activity of heterozygous and homozygous female fish compared to that of female wt fish was observed during motivated swimming trials. There is no change in the activity fraction of male mutants. The activity fraction is normalised to the time period of recording the activity of the fish, which is 30 s for motivated swimming. (D,E) Reduction in the propulsion efficiency of heterozygous and homozygous mutant males and females compared to that of wt males and females in motivated (D) and free-swimming (E) trials. Propulsion efficiency is defined as the number of bends produced by the fish per distance covered in a given time period. *N*=59 (female wt), 150 (female +/−) and 65 (female −/−), and *N*=46 (male wt), 91 (male +/−) and 84 (male −/−), in free-swimming trials; *N*=59 (female wt), 150 (female +/−) and 66 (female −/−), and *N*=45 (male wt), 96 (male +/−) and 81 (male −/−), in motivated swimming trials; and female *N*=45 (female wt), 91 (female +/−) and 57 (female −/−), and male *N*=46 (male wt), 60 (male +/−) and 75 (male −/−), in counter-current trials. Statistical significance was tested using Fisher's permutation test. *P<0.05, **P<0.01, ***P<0.001.

To examine whether mutant fish exhibit reduced endurance, we replicated the natural environmental conditions of zebrafish, whereby they swim against the water currents (Arunachalam et al., 2013; Parichy, 2015; Spence, 2011). Our experimental setup enabled fish to swim against an incoming water stream, strongest in the centre and weaker along the sides. In the two-dimensional heat map with the marginal histogram in Fig. 5A, blue represents the lowest probability density, and yellow represents the highest probability density, for the animal's presence at a given position within the tank. The results revealed that wild-type fish preferred the centre of the current. In contrast, the heterozygous mutant fish resided at an intermediate distance from the centre of the stream. Homozygous fish positioned themselves even further away from the centre of the stream, confirming that they possess less endurance and cannot constantly swim against the water current (Fig. 5A,B; Fig. S11A-C). This endurance reduction was further corroborated by observations during motivated swimming trials: *tomm70* mutant fish, particularly females, were unable to maintain constant swimming for 30 s, with most ceasing activity after ∼15-20 s (Fig. 5C).

Upon further analysis, a notable decrease in the propulsion efficiency emerged when comparing heterozygous and homozygous mutants to their wild-type counterparts. The primary decrease in propulsion efficiency was observed in heterozygous fish, with only a minimal additional decrease in the homozygous fish. This decline in propulsion efficiency was consistently observed across different test conditions, encompassing free and motivated swimming trials, as depicted in Fig. 5D,E. The disparity in propulsion efficiency was more pronounced in male specimens, a finding that aligns with the known sexual dimorphism in swimming velocities, as detailed in Garg et al. (2022). Propulsion efficiency, in this context, is quantified as the forward movement achieved per degree of body bending. Forward movement of the fish's body is intrinsically linked to the extent of its body bending (Gray, 1933; Lauder and Tytell, 2005; Muller et al., 2000; Müller and Van Leeuwen, 2006; Smits, 2019; Wardle et al., 1995). The observed decrease in propulsion efficiency in mutant fish thus suggests a fundamental alteration in their locomotive mechanics compared to that of wild types. Mutant fish have to bend their body more in order to move forward at the same speed as wild types, resulting in reduced propulsion efficiency and endurance.

### Tomm70^Ile525Thr^ mutation affects the Mauthner cell-generated C-start escape response

In order to investigate potential differences in locomotion behaviour between mutant *tomm70* fish and wild-type controls, we further compared the well-known C-start escape response between the two. The giant Mauthner cells facilitate this escape response, allowing the fish to rapidly flee from potential harm (Eaton et al., 1977; Faber and Korn, 1978; Korn and Faber, 2005; Zottoli and Faber, 2000). Notably, these large-calibre neurons and other related cells produce high-amplitude action potentials, the field potentials of which can be detected by electrodes placed in the fish tank (Issa et al., 2011).

**Fig. 6.**
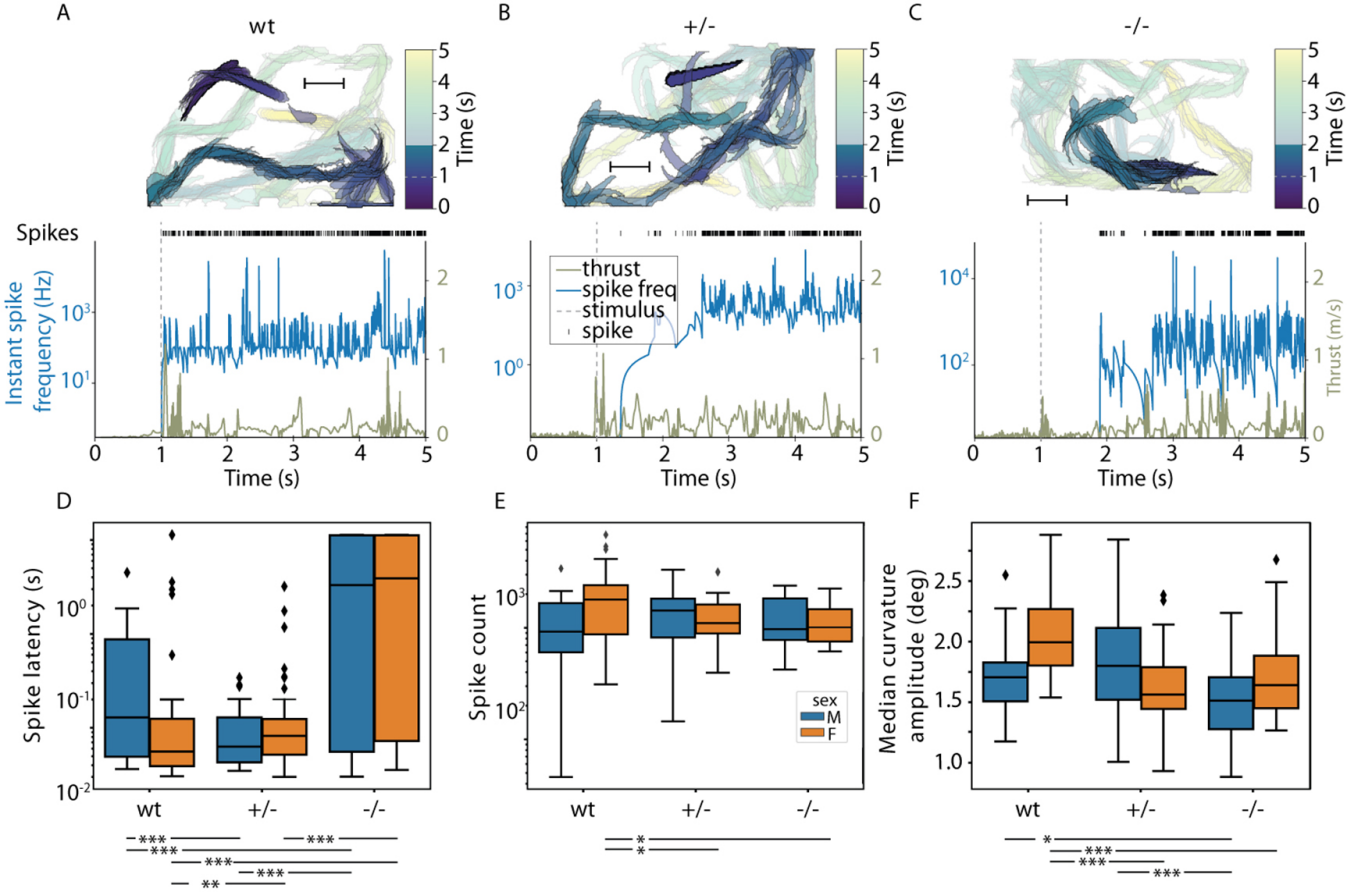
**Impact of Tomm70^Ile525Thr^ mutation on C-start escape response dynamics.** (A-C) The progression of movement traces and associated kinematic parameters for wt (A), heterozygous (B) and homozygous (C) mutant female zebrafish are illustrated during electrophysiological assessments. The top row presents sequential outlines of the zebrafish at 25 ms intervals, with the colour gradient corresponding to the adjacent colour bar. To emphasise the critical segment of the response, the 2 s window surrounding stimulus delivery (indicated by the grey dashed line on the colour bar) is rendered with increased opacity compared to the period 1-4 s after stimulus delivery. Scale bars: 10 mm. In the bottom row, large neural activity spikes are represented in a raster plot format, and the thrust magnitude and instantaneous spike frequency are depicted in the line graph beneath. The point of stimulus initiation is consistently marked by a grey dashed line for temporal orientation. (D-F) Box plots summarise spike latency (D), spike count (E) and median curvature amplitude (F). Black line denotes the median, the box denotes the interquartile range, whiskers extend to 1.5 times the interquartile range, and diamonds mark outliers. (D) Marked escalation in spike latency in heterozygous and homozygous female mutants relative to their wt female counterparts, with homozygous mutants displaying greater latency than heterozygous mutants. Analogous trends are noted in male mutants, with a notable reduction in spike latency for heterozygous males versus wt males. (E) Decrement in spike count for heterozygous and homozygous female mutants compared to that of female wt fish. (F) Reduction in median curvature amplitude for mutant females, with similar patterns observed in mutant males compared to that of their wt heterozygous counterparts. *N*=49 (female wt), 94 (female +/−) and 58 (female −/−), and *N*=42 (male wt), 56 (male +/−) and 74 (male −/−). Statistical assessments were performed using Fisher's permutation test. *P<0.05, **P<0.01, ***P<0.001.

Wild-type fish exhibited an immediate pivot and surge in speed (Fig. 6A; Fig. S12A). This instantaneous reaction was visually captured as a widely spaced silhouette trajectory, denoting swift movement away from the perceived threat (Fig. 6A). In contrast, the heterozygous mutants displayed a more subdued response, and the silhouettes appeared more closely clustered (Fig. 6B; Fig. S12B). The trajectory of homozygous mutants lacks the distinct sharp angulation associated with the C-start, suggesting a compromised or entirely absent reflexive response to the stimuli (Fig. 6C; Fig. S12C).

The discernible delay in orientation alteration and the less abrupt acceleration profile of mutants (Fig. 6B,C, bottom) are accompanied by an increased neuronal stimulus latency (Fig. 6D) and a reduction in the number of field potentials (Fig. 6E). Heterozygous fish exhibited a subtle phenotype in spike count and latency compared to that of homozygous fish. The slower and less pronounced neuronal activity of mutants correlated with a significant decrease in their bending ability (Fig. 6F), which is crucial for the C-start response. These findings suggest that the altered physiology observed in mutant *tomm70* fish, as evidenced by the BiFC assay (Fig. S3) and immunocytochemistry (Fig. 1), not only impacts the neurons themselves (Fig. 4) but also directly influences locomotion behaviour (Fig. 5) and its neuronal control, as demonstrated by the changes in the flight response (Fig. 6).

### Intact skeletal structure in *tomm70* mutants

To further substantiate that the locomotion defects in the *tomm70* mutants stem from neuronal origins rather than skeletal deformations, we utilised micro-computed tomography (CT) scanning to conduct a comprehensive examination of the vertebral columns in wild-type and *tomm70* mutant fish. Our analysis revealed no discernible differences in skeletal structure between wild types and heterozygous or homozygous mutants, regardless of sex (Fig. S13A,B). Additionally, we investigated the muscle area in the caudal region of the fish body. However, no difference was detected between wild-type, heterozygous and homozygous fish, suggesting absence of muscular atrophy in the caudal region of the mutant fish body (Fig. S14A,B). Taken together, our findings support the notion that the locomotion defects in *tomm70* mutants arise from neuronal dysfunctions.

## DISCUSSION

Tomm70 is an outer mitochondrial membrane transport protein principally involved in mediating the uptake of proteins into the mitochondria (Kreimendahl and Rassow, 2020) and interacts with a sterol-transporter ER protein called Lam6 at the ER-MCS (Murley et al., 2015). A mutation in the C-terminus of Tomm70, which results in a conserved amino acid change from isoleucine to threonine in zebrafish (Tomm70^Ile525Thr^; Fig. S1A-C), has been identified in a human patient (TOMM70^Ile554Phe^) exhibiting similar symptoms as those documented here, such as movement disorders and spasticity (Dutta et al., 2020). The absence of any impact on the structural conformation of the Tomm70 protein (Fig. S2A-C), but instead impact on its abundance (Fig. S1D-G), suggests that the mutation's consequences are functional rather than structural.

Using the BiFC assay, we could confirm that the mutation affects the interaction of Tomm70 with Lam6 specifically, as the interaction of Tomm70 with Tomm40 is unaffected (Fig. S3C-F). As Lam6 functions as a sterol transporter (Murley et al., 2015), we hypothesise that loss of the interaction of Tomm70 with Lam6 impacts cholesterol abundance in cholesterol-rich organs of the mutant fish. A significant reduction in cholesterol abundance was observed in the opaque eggs produced by the *tomm70* mutants (Fig. S3H). The observed anomalies suggest that a dysfunction in yolk cholesterol processing at stage IV of oogenesis interrupts the maturation process of oocytes, impeding the typical progression to a transparent state and leading to sustained opacity (Dosch et al., 2004).

Lipid metabolism disturbances, along with impaired transport of critical components such as RNA, proteins and organelles to the axon and axon terminal, are known to contribute to neurodegeneration (Guo et al., 2020; Liu et al., 2012). Among the organelles, mitochondria play a crucial role in ATP and metabolite production, thereby regulating cellular energy levels and metabolic pathways. Impaired mitochondrial transport machinery can lead to progressive neuronal degeneration (Guo et al., 2020; Liu et al., 2012; Maday et al., 2014; Saxton and Hollenbeck, 2012).

In wild-type neurons, Tomm70 is incorporated into the outer mitochondrial membrane (Eaglesfield and Tokatlidis, 2021), and these mitochondria can freely travel into the axon and its terminals. In fish carrying the mutant Tomm70^Ile525Thr^, mitochondria that incorporate the mutant protein appear to be retained near the soma and are unable to reach the axon terminals. However, in mutant neurons, mitochondria that do not incorporate any Tomm70 protein can still be transported freely (Fig. 2A-C). This is likely because the overall abundance of Tomm70 is significantly reduced in mutants, resulting in many mitochondria lacking Tomm70 altogether.

The absence of Tomm70 in mitochondria transported to axons could profoundly impact their function, as Tomm70 is crucial for importing numerous essential proteins into mitochondria (Kreimendahl and Rassow, 2020). Our data suggest a possible sexual dimorphism in this phenotype, warranting further investigation. The phenotype in females (Fig. 1) seems to be more pronounced than that in males (Fig. S7). Additionally, Tomm70 is part of the ER-MCS, facilitating the transport of sterols and Ca2+ into mitochondria (Filadi et al., 2018; Murley et al., 2015), and its absence could disrupt these processes. Furthermore, dendritic mitochondria are essential for maintaining synapses (Li et al., 2004), and the absence of Tomm70 could contribute to synaptic instability. As the mutation does not appear to affect the protein's structural conformation, further investigation is needed to determine how the mutation influences the transport of mitochondria to axons and dendrites and identify the processes most affected by this alteration.

Absence of Tomm70 protein from the distal parts of long axons can affect the downstream cellular processes for which the supply of Tomm70 or mitochondria is essential. One such process is the formation and maintenance of myelin sheath surrounding the large calibre axons. Myelin pathology and demyelination are common features of the degeneration of neurons in many neurodegenerative disorders (Weil et al., 2016). Although myelin composition in zebrafish differs from that in other vertebrates (Siems et al., 2021), our findings reveal that prominent signs of pathology, such as splitting and vesiculation of myelin sheath, were more frequently observed in large-calibre spinal cord axons of *tomm70* mutants than in those of wild types (Fig. 4B-F). This suggests that the integrity of the myelin sheath is affected in the absence of Tomm70. Given the essential role of mitochondria in oligodendrocyte function and myelin sheath development (Meyer and Rinholm, 2021), our observations suggest that the myelin pathology and absence of Tomm70 in axons are functionally related.

The physiological changes induced by the mutation in *tomm70* mutants had notable effects on their motor behaviour. The *tomm70* mutants required substantially greater bending to generate thrust velocities comparable to wild-type fish (Fig. 5D,E), suggesting a more energetically demanding locomotion that contributes to their diminished endurance and premature cessation of flight behaviour (Fig. 5B,C). The mutation also affects the Mauthner cell-governed C-start escape response. Electrophysiological analysis of the neuronal activity during C-starts revealed that there is an increase in the latency of the field potentials and a reduction in the number of spikes (Fig. 6D,E). Activating the escape response revealed that *tomm70* mutant fish are less able to bend their bodies than their wild-type counterparts (Fig. 6F). Heterozygous mutants display intermediate locomotion phenotypes compared to homozygous fish, but the latency during C-starts is ten times higher in homozygous fish, suggesting a greater impact of the mutation on Mauthner cells than on other motor neurons. As Mauthner cells are considered functional homologues of the mammalian corticospinal tract (Davis and Farel, 1990; Will, 1986), these findings suggest a potential evolutionary link between the two systems. Furthermore, our results indicate that the mutation not only affects the motor neurons themselves but also directly influences the locomotion activity governed by them, highlighting the mutation's impact on both neuronal structure and function.

Our analysis revealed no significant differences in the vertebral column anatomy between wild-type, heterozygous and homozygous *tomm70* mutant fish (Fig. S13A,B), confirming that the alterations in locomotion behaviour stem from motor neuron degeneration rather than skeletal deformities. Additionally, the absence of significant caudal muscle area reduction in *tomm70* mutants (Fig. S14A,B) further implies that the behavioural phenotypes are likely rooted in neuronal control factors.

## Conclusions

By examining the effects of a point mutation in the ER-MCS protein Tomm70 across multiple biological layers in zebrafish, our study provides insights into potential mechanisms of motor neuron dysfunction. In light of these findings, the report of neurodegenerative symptoms in a human patient with an identical variant (TOMM70^Ile554Phe^; Dutta et al., 2020) and the previous prediction of *tomm70* as a potential causal gene for HSP (Novarino et al., 2014), we propose its inclusion in genetic screenings to improve the early detection and diagnosis of neurodegenerative disorders.

## MATERIALS AND METHODS

### Zebrafish husbandry

Zebrafish were maintained according to the guidelines provided by the Westerfield zebrafish book (Westerfield, 2000) and EuFishBioMed/Federation of European Laboratory Animal Science Associations (FELASA) (Aleström et al., 2020), in compliance with the regulations of Georg-August-University Göttingen and Bielefeld University, Bielefeld, Germany (540.4/16 25/Engelmann). The zebrafish experiments were approved by the Lower Saxony State Office for Consumer Protection and Food Safety (AZ14/1681/Dosch) and conducted according to European Union directive 2010/63/EU.

The *ruehrei^p25ca^* mutant we analysed in this study was originally identified through a maternal-effect mutant screen in zebrafish (Dosch et al., 2004). We identified this mutant as Tomm70^Ile525Thr^ by RNA sequencing. Another mutant from this screen, *souffle*, exhibiting an identical egg phenotype, was found to carry a mutation in the *spastizin* (also known as *zfyve26*) gene (Kanagaraj et al., 2014). Prior research in our laboratory showed that neurodegenerative symptoms in *spastizin* mutants only manifest in late-stage adults (Garg et al., 2024). Consequently, we conducted our study on Tomm70^Ile525Thr^ adult fish aged 12 to 18 months to capture a more accurate assessment of these late-onset symptoms.

### mRNA sequencing

We extracted the total RNA from the ovary of wild-type and *ruehrei^p25ca^* mutant fish. The mRNA was enriched by selecting poly(A) tails using oligo(dT) beads, thereby removing ribosomal RNA and mitochondrial RNA. Once mRNA was isolated, it was converted into sequencing libraries using a TruSeq RNA Library Prep Kit v2 (Illumina, Germany) and subjected to sequencing using an Illumina HiSeq 2000. Sequence images were transformed with Illumina software BaseCaller to base call (BCL) files, which were demultiplexed to fastq files with consensus assessment of sequence and variation (CASAVA) (v1.8.2). Subsequently, reads were aligned by spliced transcripts alignment to a reference (STAR) (2.3.9e) (Dobin et al., 2013) to the Ensembl *Danio rerio* genome (version Zv9). Duplicates were eliminated and single-nucleotide polymorphism (SNP) piled up by samtools (0.1.19). VarScan (v2.3.6) (Koboldt et al., 2012) was applied for SNP calling and limiting SNP areas. Data underwent pre-processing and analysis within the R/Bioconductor environment (3.0.2/2.14). Mutation effects were identified via the Ensembl variant effect predictor (McLaren et al., 2010).

### Bioinformatics methods

Multiple sequence alignment was performed using Clustal Omega program (Sievers et al., 2011). The I-TASSER server was used to predict the structure of wild-type and mutant Tomm70 protein (Yang and Zhang, 2015; Zhang et al., 2017; Zhou et al., 2022). The TM-align server of I-TASSER (Zhang and Skolnick, 2005) and PyMOL application were used to align the predicted model for mutant and wild-type Tomm70.

### Western blotting

Whole-brain samples from each genotype were prepared for western blotting by homogenising the tissue in a mix of protease inhibitor (Thermo Fisher Scientific, Germany) and radio-immunoprecipitation assay (RIPA) buffer [0.1% sodium dodecyl sulphate (SDS; Serva Electrophoresis, Germany), 1% Nonidet P-40 (NP40; Merck/Sigma-Aldrich, Germany), 1% sodium deoxycholate (Merck/Sigma-Aldrich), 150 mM sodium chloride (NaCl; Carl Roth, Germany), 50 mM Tris (hydroxymethyl) aminomethane (THAM) hydrochloride (Tris-HCl; pH 7.2-7.5; Carl Roth)] in a ratio of 1:100. The lysates were centrifuged at 10,000 ***g*** for 10 min at 4°C. The supernatant was collected, and samples were subjected to Bradford assay for estimating the protein concentration (Bradford, 1976). Then, 30 µg lysate from each sample type was separated on a 8% SDS-PAGE gel at 30 mA for 1-1.5 h and blotted on nitrocellulose membrane (Bio-Rad, Germany) at 180 mA for 1.5 h. The membrane was blocked for 1 h at room temperature in 4% powdered milk (Carl Roth) in phosphate buffered saline (PBS; ChemSolute, Germany) with 0.1% Tween (AppliChem, Germany) (PBST). For this study, anti-β-Tubulin antibody was used as the loading control because Tomm70 and β-Tubulin are nearly in the same size range, and the available antibodies were both raised in mouse. We incubated the blot with anti-Tomm70 primary antibody (1:250 or 1:300; Proteintech, Germany) in 4% milk in PBST overnight at 4°C, and then horseradish peroxidase (HRP) anti-mouse secondary antibody (1:2500; Invitrogen, Germany) in 4% milk in PBST for 30 min at room temperature. After capturing the images for the Tomm70 signal, the membrane was washed with PBST for 5 min and then with stripping buffer [0.5 M NaOH (AppliChem, Germany) in water] for 5 min. It was followed by two more washes with PBST for 5 min each. The membrane was again blocked for 1 h in 4% milk in PBST, followed by incubation with anti-β-Tubulin (1:1000; Developmental Studies Hybridoma Bank, USA) and HRP anti-mouse secondary antibody (1:2500; Invitrogen) for 30 min each. Images were captured and analysed using iBright CL1000 (Invitrogen) and ImageJ/FIJI (Schindelin et al., 2012), respectively.

### BiFC assay

The investigation of Tomm70 interactions with Lam6 and Tomm40 was conducted using the BiFC assay (Kerppola, 2006, 2008). In this study, Venus served as the fluorescent reporter protein. Its N-terminal half (VN) was fused to both the N- and C-termini of Tomm70, whereas its C-terminal half (VC) was fused to the N- and C-termini of Tomm40 and Lam6. All fusion constructs were generated using either the Gateway or In-Fusion cloning method, as previously described (Perera and Dosch, 2021). For Tomm70 and Tomm40 fusion constructs, templates were amplified from complementary DNA, derived from the reverse transcription of ovarian RNA. The template for Lam6 was amplified from the genomic DNA of yeast.

#### Microinjection

A total of 2 nl capped sense RNA, diluted with 0.1 M KCl and 0.05% Phenol Red (Merck/Sigma-Aldrich), was injected into one-cell-stage zebrafish embryos using PV820 WPI injecting apparatus (Sarasota, USA). Injected embryos were raised at 28°C in E3 medium (5 mM NaCl, 0.17 mM KCl, 0.33 mM CaCl_2_ and 0.33 mM MgSO_4_). The injected embryos were sorted and manually counted and imaged 7-8 h post fertilisation for fluorescence using the green fluorescent protein (GFP) channel of a fluorescence stereomicroscope (Lumar V12, Zeiss, Germany). Images were processed using ImageJ/FIJI (Schindelin et al., 2012).

### Lipid extraction and mass spectrometry

The transparent and opaque eggs produced by wild-type and mutant zebrafish were flash frozen in liquid nitrogen in pre-weighed Eppendorf cups and stored at −80°C until further processing. The weight of samples was determined after lyophilisation. Lyophilised samples were ground in a ball mill to make a fine powder. For the two-phase extraction process, 400 µl methyl-tert-butyl ether [methanol (3:1; v/v; all solvents were high-performance liquid chromatography grade (Thermo Fisher Scientific)] was added, followed by vortexing and addition of 200 µl ultra-pure water. As an internal standard, 5 µg 17:0 free fatty acid (Merck, Germany) was added, and samples were extracted for 30 min. The samples were then centrifuged at maximum speed (21,130 ***g***) for 1 min, and the resulting upper phase was transferred to a new tube and stored at −20°C until further processing. For GC-MS measurements, 20-50 µl of the upper phase was evaporated under a stream of nitrogen, redissolved in 10-15 µl anhydrous pyridine (Merck) and derivatised with twice the volume of N-methyl-N-trimethylsilyltrifluoroacetamid (Merck) to yield trimethylsilylated analytes. Samples were analysed on an Agilent 7890B gas chromatograph connected to an Agilent 5977N mass-selective detector, as described (Berghoff et al., 2021). Cholesterol was identified in comparison to an external standard. For quantification, GC/MSD Mass Hunter software with MSD ChemStation Data Analysis (Agilent Technologies) was used. For the internal standard and cholesterol, the mass-to-charge ratios 327 Da/e and 458 Da/e, respectively, were quantified as target ions. Values for cholesterol were normalised to the internal standard and the mass of the sample.

### Cell culture

#### Primary culture of zebrafish brain neurons

The protocol for primary neuronal cultures for zebrafish brain was adapted from studies with insect neurons (Knorr et al., 2020, 2022). One whole brain per culture from wild-type and *tomm70* mutants was dissected and collected in Leibovitz 15 (L-15) medium (Gibco, Life Technologies, Germany) supplemented with 1% penicillin/streptomycin (Merck/Sigma-Aldrich). Then, the samples were enzymatically digested with collagenase/dispase (2 mg/ml; Merck/Sigma-Aldrich) for 30 min at 27°C. The enzymatic reaction was stopped by replacing the solution with Hank's balanced salt solution (Thermo Fisher Scientific). The brain stem was triturated in the Hank's solution and centrifuged for 3 min (2191 ***g***), after which a pellet was formed. After removal of supernatant, the pellet was resuspended in L-15 medium, and the cell suspension was seeded on a concanavalin A (Merck/Sigma-Aldrich)-coated coverslip and incubated for 1.5 h. Subsequently, culture dishes were filled with L-15 medium and supplemented with 4% foetal bovine serum gold (PAA Laboratories GmbH, Austria). Medium was changed every second day for ∼2 weeks until the neurons developed long axons. The cultures were maintained at 27°C without CO_2_ buffering.

#### Transfection

Human embryonic kidney (HEK) cells were grown in Dulbecco's modified Eagle medium (DMEM; Thermo Fisher Scientific) supplemented with 10% foetal calf serum (DMEM+; Merck/Sigma-Aldrich) and split when the confluence reached ∼95%. Old medium was removed by aspiration, and cells were washed with 1 ml 1× PBS. To detach the cells, 750 µl 1× trypsin (Thermo Fisher Scientific) was added. Detached cells were washed with 5 ml DMEM+ and pelleted down at 300 ***g*** for 5 min at 24°C. Pelleted cells were resuspended in 1 ml fresh DMEM+, and 200-250 µl was seeded in a new flask. For transfection, 70-100 µl of the cells was seeded on concanavalin A (Merck/Sigma-Aldrich)-coated coverslips and filled with 1.4 ml DMEM+. An Effectene transfection kit (Qiagen, Germany) was used to transfect the cells with mCherry-Mito-7 (Addgene #55102) plasmid. The transfection mix was prepared by adding 94 µl EC-buffer, 500 ng plasmid and 4 µl enhancer for one well of a six-well plate. The compounds were mixed by vortexing for 5 s and incubated for 5 min at room temperature. Then, 5 µl Effectene was added and mixed by flipping the vial manually, followed by incubation for 8-10 min at room temperature. This mixture was then added to the cells before incubating at 37°C with 5% CO_2_. HEK cells were seen to express the protein within 24-48 h.

#### Immunocytochemistry

As neuronal development progressed in the primary culture, cells were fixed with 400 µl 4% paraformaldehyde (PFA; Carl Roth) for 30 min. Unless otherwise stated, all washes were for 5 min each. Coverslips were first washed with PBS three times, then five times with PBST [PBS containing 0.1% Triton X-100 (Merck/Sigma-Aldrich)]. Cells were blocked with 300 µl blocking solution [5% normal goat serum (NGS; Jackson ImmunoResearch, UK), 0.25% bovine serum albumin (BSA; Carl Roth) in PBS containing 0.3% Triton-X 100] for 1 h at room temperature. Subsequently, the cells were incubated with anti-Cytochrome c antibody (1:450; Proteintech) in blocking solution overnight at 4°C. After washing coverslips five times with PBST and three times with PBS, the cells were incubated with goat anti-mouse Alexa Fluor 633 (1:1500; Invitrogen) and 4′,6-diamidino-2-phenylindole (DAPI; 1:1000; Merck/Sigma-Aldrich) in PBST for 1 h at room temperature. After five washes with PBS, the cells were stained with CoraLite Plus 488-conjugated anti-β-Tubulin (1:450 or 1:500; Proteintech) and CoraLite Plus 594-conjugated anti-Tomm70 (1:400; Proteintech) primary antibodies for 3-4 h at 4°C. Then, again after washing, coverslips were flipped upside down and mounted with 1,4-diazabicyclo[2.2.2]octane (DABCO; Carl Roth), and slides were stored at 4°C. As anti-β-Tubulin and anti-Tomm70 antibodies are directly conjugated to fluorophore, for staining neurons with only these two antibodies, the protocol remained the same, except for adding no secondary antibodies but only DAPI after the primary antibody.

To confirm the specificity of the anti-Cytochrome c antibody, coverslips with HEK cells were transferred to four-well plates and fixed with 300 µl 4% PFA for 10 min. After washing cells three times with PBS and then twice with PBST, they were blocked with blocking solution (5% NGS, 0.25% BSA in PBS containing 0.3% Triton-X 100) for 1 h at room temperature. Following this, cells were incubated with anti-Cytochrome c antibody (1:300; Proteintech) in blocking solution for 30 min at 4°C and washed twice with PBST and twice with PBS. Next, cells were incubated with secondary goat anti-mouse Alexa Fluor 488 (1:1500; Life Technologies, Germany) and DAPI (1:1000) in PBST for 30 min at room temperature. After three washes with PBS, coverslips were flipped upside down and mounted with DABCO, and slides were stored at 4°C. In the case of the anti-Cytochrome c and mCherry-Mito-7 co-staining, we could directly observe the signal overlap and thereby confirm the specificity of our anti-Cytochrome c antibody (Fig. S6A). CoraLite Plus 594-conjugated anti-Tomm70 antibody has a similar emission range to mCherry, so to make sure that we saw a true signal for this antibody, we quenched the original mCherry signal by fixing cells in 100 µl more 4% PFA and for 5-10 more min. The rest of the procedure was the same as for anti-Cytochrome c antibody, until the blocking step. After blocking, cells were incubated with CoraLite Plus 594-conjugated anti-Tomm70 (1:400; Proteintech) and Fluo Tag X4 anti-red fluorescent protein (RFP), Alexa Fluor 647 (1:150; NanoTag, Germany) (to recognise the mCherry) antibodies. This process led to an overlay of the anti-mCherry signal with the signal from anti-Tomm70 antibody (Fig. S6B). The distinct overlay of the different fluorescent signals confirmed the accuracy of our antibody targeting, thus validating our experimental approach.

#### Confocal microscopy

Images of primary brain neurons and HEK cells were acquired using a TCS Sp8 confocal microscope (Leica, Germany) with 63× glycerol immersion objective and scanning resolution of 1024×1024 pixels. Axons positive for the Tomm70 and Cytochrome c signal were counted manually, and the images were processed using ImageJ/FIJI (Schindelin et al., 2012).

### High-pressure freezing and electron microscopy

After sacrificing zebrafish, the spinal cord was dissected and separated into three segments using the number of vertebrae for orientation. A 3 mm-long segment of the upper and lower part of the cord was cryofixed in 20% polyvinylpyrrolidone (molecular mass 10,000 g/mol) (Sigma-Aldrich, Germany) in PBS, using a Leica HPM100 high-pressure freezer. The sections were then freeze substituted using a Leica AFS2 and Epon (Serva Electrophoresis) embedded, as previously described (Weil et al., 2019). Then, 0.5 µm semi-thin or 50 nm ultra-thin sections of the embedded tissue were cut using a Leica UC7 ultramicrotome, which were contrasted with UranylLess stain (Science Services GmbH, Germany). Electron micrographs were acquired using a Zeiss LEO EM912AB equipped with a wide-angle dual speed 2k-charged-coupled device camera (TRS, Germany). Images were marked manually for signs of demyelination using the cell counter feature of ImageJ/FIJI.

### Locomotion recordings

Genie HM1024 high-speed camera (Dalsa Imaging Solutions GmbH, Germany) linked with an Optem Zoom 125C 12.5:1 Micro-Inspection Lens System was used to record the movement of zebrafish from above. A light-emitting diode light plate (Lumitronix, Germany) and an aquarium light control (Elektronik-Werkstatt SSF, University of Göttingen, Göttingen, Germany) were used for illuminating the setup from below. Recordings were conducted between 10:00 and 20:00 in the diurnal rhythm.

#### Free and motivated swimming trials

The experiments for free and motivated swimming trials were conducted in a 24.9×11.4 cm acrylic glass aquarium with a shallow water depth of 1.6 cm. Videos for these experiments were recorded for 30 s at 200 frames/s. The recording for cruising started 30 s after transferring the fish to the setup tank. The motivated trial started directly after cruising recording ended by tossing a 474 g metal weight through a plastic tunnel, which struck the setup table and created a mechanical stimulus of 18.7 N on the surface.

#### Counter-current trials

To generate a constant water flow, we used a custom-built 17.2×4.4 cm Plexiglas^®^ aquarium fitted with an installed water pump, running at 150 ml/s. The movement of fish against the water stream was recorded for 30 s at a frame rate of 501 frames/s.

#### Electrophysiology

To measure the electric field potential from the neurons of freely behaving zebrafish, we adapted the experimental setup from Issa et al. (2011). The tank used for this study was 8×4×4 cm. The escape response of the fish was evoked using the water jet produced by a picospritzer pressure pump (Parker Hannifin, USA). The electric signal was amplified 2000 times and band-pass filtered with a pass window of 300-500 Hz, and 50 Hz noise was additionally filtered out using a Hum Bug (Quest Scientific, USA). Milli-Q water was used to achieve a resistance of 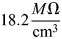. The filtered and amplified electric signals were recorded using a micro2 1401 (Cambridge Electronic Design, UK) data acquisition system and subsequently analysed with Spike2 software (Cambridge Electronic Design). The picospritzer pump triggered the video camera, and the movement of animal in response to the water jet was recorded for 5 s at a frame rate of 923 frames/s.

#### Behavioural data analysis

Limbless Animal traCkEr (LACE), a MATLAB R2012b script (MathWorks, USA), was used to track the animals (Garg et al., 2022).

### Micro-CT scan

After sacrificing and briefly rinsing in water, zebrafish were transferred to 35% and 70% ethanol for 1 h each. For staining and fixation, fish were placed for ∼10 days at room temperature under slow rotation in a 4% PFA solution (Serva Electrophoresis) in PBS, pH 7.4 (Invitrogen), containing 0.7% phosphotungstic acid solution (Merck/Sigma-Aldrich) diluted in 70% ethanol. For storage, fish were briefly rinsed in water and embedded in 1% agarose (Carl Roth) in 1.8 ml vials (Nunc CryoTube Vials, Merck/Sigma-Aldrich). The specimens were scanned with an *in vivo* micro-CT system Quantum FX (Perkin Elmer, USA) operated with the following settings: tube voltage, 90 kV; tube current, 200 µA; field of view (FOV), 10×10 mm (Arunachalam et al., 2013); total acquisition time, 3 min; resulting in a reconstructed pixel size of 20 µm and an image matrix of 512×512×512 voxel (Dullin et al., 2017). Two to three consecutive scans were acquired and stitched together using a custom-made Python script that, in addition to finding the perfect overlap, also corrects for drift in between the scans. Data were analysed using ImageJ/FIJI (Schindelin et al., 2012).

### Statistics

Independent *t*-test (William, 1908) and Wilcoxon rank-sum test (Mann and Whitney, 1947) were used to calculate the statistical significance for western blot and cholesterol measurements, respectively. For all other datasets, Fisher's permutation test (Crowley, 1992; Ernst, 2004; Fisher, 1970) was used, in which samples were bootstrapped 200,000 times. All *P*-values were corrected with Benjamini–Hochberg false discovery rate correction (Benjamini and Hochberg, 1995).

## Supplementary Material

10.1242/dmm.052029_sup1 Supplementary information

## References

[DMM052029C1] Aleström, P., D'Angelo, L., Midtlyng, P. J., Schorderet, D. F., Schulte-Merker, S., Sohm, F. and Warner, S. (2020). Zebrafish: housing and husbandry recommendations. *Lab. Anim.* 54, 213-224. 10.1177/0023677219869037 31510859 PMC7301644

[DMM052029C2] Arunachalam, M., Raja, M., Vijayakumar, C., Malaiammal, P. and Mayden, R. L. (2013). Natural history of zebrafish (danio rerio) in india. *Zebrafish* 10, 1-14. 10.1089/zeb.2012.0803 23590398

[DMM052029C3] Benjamini, Y. and Hochberg, Y. (1995). Controlling the false discovery rate: a practical and powerful approach to multiple testing. *J. R. Stat. Soc. B Methodol.* 57, 289-300. 10.1111/j.2517-6161.1995.tb02031.x

[DMM052029C4] Berghoff, S. A., Spieth, L., Sun, T., Hosang, L., Schlaphoff, L., Depp, C., Düking, T., Winchenbach, J., Neuber, J., Ewers, D. et al. (2021). Microglia facilitate repair of demyelinated lesions via post-squalene sterol synthesis. *Nat. Neurosci.* 24, 47-60. 10.1038/s41593-020-00757-6 33349711 PMC7116742

[DMM052029C5] Blackstone, C. (2012). Cellular pathways of hereditary spastic paraplegia. *Annu. Rev. Neurosci.* 35, 25-47. 10.1146/annurev-neuro-062111-150400 22540978 PMC5584684

[DMM052029C6] Blackstone, C. (2018). Hereditary spastic paraplegia. *Handb. Clin. Neurol.* 148, 633-652. 10.1016/B978-0-444-64076-5.00041-7 29478605

[DMM052029C7] Bradford, N. (1976). A rapid and sensitive method for the quantitation microgram quantities of a protein isolated from red cell membranes. *Anal. Biochem.* 72, e254. 10.1016/0003-2697(76)90527-3 942051

[DMM052029C8] Crowley, P. H. (1992). Resampling methods for computation-intensive data analysis in ecology and evolution. *Annu. Rev. Ecol. Syst.* 23, 405-447. 10.1146/annurev.es.23.110192.002201

[DMM052029C9] Davis, G., Jr and Farel, P. B. (1990). Mauthner cells maintain their lumbar projection in adult frog. *Neurosci. Lett.* 113, 139-143. 10.1016/0304-3940(90)90293-I 2377313

[DMM052029C10] Dobin, A., Davis, C. A., Schlesinger, F., Drenkow, J., Zaleski, C., Jha, S., Batut, P., Chaisson, M. and Gingeras, T. R. (2013). Star: ultrafast universal rna-seq aligner. *Bioinformatics* 29, 15-21. 10.1093/bioinformatics/bts635 23104886 PMC3530905

[DMM052029C11] Dosch, R., Wagner, D. S., Mintzer, K. A., Runke, G., Wiemelt, A. P. and Mullins, M. C. (2004). Maternal control of vertebrate development before the midblastula transition: mutants from the zebrafish i. *Dev. Cell* 6, 771-780. 10.1016/j.devcel.2004.05.002 15177026

[DMM052029C12] Dullin, C., Ufartes, R., Larsson, E., Martin, S., Lazzarini, M., Tromba, G., Missbach-Guentner, J., Pinkert- Leetsch, D., Katschinski, D. M. and Alves, F. (2017). µct of *ex-vivo* stained mouse hearts and embryos enables a precise match between 3d virtual histology, classical histology and immunochemistry. *PLoS ONE* 12, e0170597. 10.1371/journal.pone.0170597 28178293 PMC5298245

[DMM052029C13] Dutta, D., Briere, L. C., Kanca, O., Marcogliese, P. C., Walker, M. A., High, F. A., Vanderver, A., Krier, J., Carmichael, N., Callahan, C. et al. (2020). De novo mutations in tomm70, a receptor of the mitochondrial import translocase, cause neurological impairment. *Hum. Mol. Genet.* 29, 1568-1579. 10.1093/hmg/ddaa081 32356556 PMC7268787

[DMM052029C14] Eaglesfield, R. and Tokatlidis, K. (2021). Targeting and insertion of membrane proteins in mitochondria. *Front. Cell Dev. Biol.* 9, 803205. 10.3389/fcell.2021.803205 35004695 PMC8740019

[DMM052029C15] Eaton, R. C., Bombardieri, R. A. and Meyer, D. L. (1977). The mauthner-initiated startle response in teleost fish. *J. Exp. Biol.* 66, 65-81. 10.1242/jeb.66.1.65 870603

[DMM052029C16] Elsayed, L. E., Eltazi, I. Z., Ahmed, A. E. and Stevanin, G. (2021). Insights into clinical, genetic, and pathological aspects of hereditary spastic paraplegias: a comprehensive overview. *Front. Mol. Biosci.* 8, 690899. 10.3389/fmolb.2021.690899 34901147 PMC8662366

[DMM052029C17] Ernst, M. D. (2004). Permutation methods: a basis for exact inference. *Stat. Sci.* 19, 676-685. 10.1214/088342304000000396

[DMM052029C18] Faber, D. and Korn, H. (1978). Electrophysiology of the Mauthner cell: basic properties, synaptic mechanisms, and associated networks. *Neurobiol. Mauthner Cell* 47-131.

[DMM052029C19] Filadi, R., Leal, N. S., Schreiner, B., Rossi, A., Dentoni, G., Pinho, C. M., Wiehager, B., Cieri, D., Calì, T., Pizzo, P. et al. (2018). Tom70 sustains cell bioenergetics by promoting ip3r3-mediated er to mitochondria ca2+ transfer. *Curr. Biol.* 28, 369-382. 10.1016/j.cub.2017.12.047 29395920

[DMM052029C20] Fink, J. K. (2006). Hereditary spastic paraplegia. *Curr. Neurol. Neurosci. Rep.* 6, 65-76. 10.1007/s11910-996-0011-1 16469273

[DMM052029C21] Fink, J. K. (2014). Hereditary spastic paraplegia: clinical principles and genetic advances. *Semin. Neurol.* 34, 293-305. 10.1055/s-0034-1386767 25192507

[DMM052029C22] Finsterer, J., Löscher, W., Quasthoff, S., Wanschitz, J., Auer-Grumbach, M. and Stevanin, G. (2012). Hereditary spastic paraplegias with autosomal dominant, recessive, x-linked, or maternal trait of inheritance. *J. Neurol. Sci.* 318, 1-18. 10.1016/j.jns.2012.03.025 22554690

[DMM052029C23] Fisher, R. A. (1970). Statistical methods for research workers. In *Breakthroughs in Statistics: Methodology and Distribution*, pp. 66-70. New York: Springer New York.

[DMM052029C24] Garg, V., André, S., Giraldo, D., Heyer, L., Göpfert, M. C., Dosch, R. and Geurten, B. R. (2022). A markerless pose estimator applicable to limbless animals. *Front. Behav. Neurosci.* 16, 819146. 10.3389/fnbeh.2022.819146 35418841 PMC8997243

[DMM052029C25] Garg, V., André, S., Heyer, L., Kracht, G., Ruhwedel, T., Scholz, P., Ischebeck, T., Werner, H. B., Dullin, C., Engelmann, J. et al. (2024). Axon demyelination and degeneration in a zebrafish spastizin model of hereditary spastic paraplegia. *Open Biol.* 14, 240100. 10.1098/rsob.240100 39503232 PMC11539067

[DMM052029C26] Giudice, T. L., Lombardi, F., Santorelli, F. M., Kawarai, T. and Orlacchio, A. (2014). Hereditary spastic paraplegia: clinical-genetic characteristics and evolving molecular mechanisms. *Exp. Neurol.* 261, 518-539. 10.1016/j.expneurol.2014.06.011 24954637

[DMM052029C27] Gray, J. (1933). Studies in animal locomotion: I. the movement of fish with special reference to the eel. *J. Exp. Biol.* 10, 88-104. 10.1242/jeb.10.1.88

[DMM052029C28] Guo, W., Stoklund Dittlau, K. and Van Den Bosch, L. (2020). Axonal transport defects and neurodegeneration: molecular mechanisms and therapeutic implications. *Semin. Cell Dev. Biol.* 99, 133-150. 10.1016/j.semcdb.2019.07.010 31542222

[DMM052029C29] Harding, A. E. (1993). Hereditary spastic paraplegias. *Semin. Neurol.* 13, 333-336. 10.1055/s-2008-1041143 8146482

[DMM052029C30] Harding, A. E. (1984). The hereditary ataxias and related disorders. *Clin. Neurol. Neurosurg. Monographs* 6, 57-70.

[DMM052029C31] Issa, F. A., O'Brien, G., Kettunen, P., Sagasti, A., Glanzman, D. L. and Papazian, D. M. (2011). Neural circuit activity in freely behaving zebrafish (danio rerio). *J. Exp. Biol.* 214, 1028-1038. 10.1242/jeb.048876 21346131 PMC3044078

[DMM052029C32] Kanagaraj, P., Gautier-Stein, A., Riedel, D., Schomburg, C., Cerdà, J., Vollack, N. and Dosch, R. (2014). Souf- fle/spastizin controls secretory vesicle maturation during zebrafish oogenesis. *PLoS Genet.* 10, e1004449. 10.1371/journal.pgen.1004449 24967841 PMC4072560

[DMM052029C33] Kerppola, T. K. (2006). Design and implementation of bimolecular fluorescence complementation (bifc) assays for the visualization of protein interactions in living cells. *Nat. Protoc.* 1, 1278-1286. 10.1038/nprot.2006.201 17406412 PMC2518326

[DMM052029C34] Kerppola, T. K. (2008). Bimolecular fluorescence complementation (bifc) analysis as a probe of protein interactions in living cells. *Annu. Rev. Biophys.* 37, 465-487. 10.1146/annurev.biophys.37.032807.125842 18573091 PMC2829326

[DMM052029C35] Klebe, S., Stevanin, G. and Depienne, C. (2015). Clinical and genetic heterogeneity in hereditary spastic paraplegias: from spg1 to spg72 and still counting. *Rev. Neurol. (Paris)* 171, 505-530. 10.1016/j.neurol.2015.02.017 26008818

[DMM052029C36] Knorr, D. Y., Georges, N. S., Pauls, S. and Heinrich, R. (2020). Acetylcholinesterase promotes apoptosis in insect neurons. *Apoptosis* 25, 730-746. 10.1007/s10495-020-01630-4 32761307 PMC7527371

[DMM052029C37] Knorr, D. Y., Schneider, K., Büschgens, L., Förster, J., Georges, N. S., Geurten, B. R. and Heinrich, R. (2022). Pro- tection of insect neurons by erythropoietin/crlf3-mediated regulation of pro-apoptotic acetylcholinesterase. *Sci. Rep.* 12, 18565. 10.1038/s41598-022-22035-0 36329181 PMC9633726

[DMM052029C38] Koboldt, D. C., Zhang, Q., Larson, D. E., Shen, D., McLellan, M. D., Lin, L., Miller, C. A., Mardis, E. R., Ding, L. and Wilson, R. K. (2012). Varscan 2: somatic mutation and copy number alteration discovery in cancer by exome sequencing. *Genome Res.* 22, 568-576. 10.1101/gr.129684.111 22300766 PMC3290792

[DMM052029C39] Korn, H. and Faber, D. S. (2005). The mauthner cell half a century later: a neurobiological model for decision- making? *Neuron* 47, 13-28. 10.1016/j.neuron.2005.05.019 15996545

[DMM052029C40] Kreimendahl, S. and Rassow, J. (2020). The mitochondrial outer membrane protein Tom70-mediator in protein traffic, membrane contact sites and innate immunity. *Int. J. Mol. Sci.* 21, 7262. 10.3390/ijms21197262 33019591 PMC7583919

[DMM052029C41] Lauder, G. V. and Tytell, E. D. (2005). Hydrodynamics of undulatory propulsion. *Fish Physiol.* 23, 425-468. 10.1016/S1546-5098(05)23011-X

[DMM052029C42] Li, Z., Okamoto, K.-I., Hayashi, Y. and Sheng, M. (2004). The importance of dendritic mitochondria in the morphogenesis and plasticity of spines and synapses. *Cell* 119, 873-887. 10.1016/j.cell.2004.11.003 15607982

[DMM052029C43] Liu, X.-A., Rizzo, V. and Puthanveettil, S. (2012). Pathologies of axonal transport in neurodegenerative diseases. *Transl. Neurosci.* 3, 355-372. 10.2478/s13380-012-0044-7 23750323 PMC3674637

[DMM052029C44] Maday, S., Twelvetrees, A. E., Moughamian, A. J. and Holzbaur, E. L. (2014). Axonal transport: cargo-specific mechanisms of motility and regulation. *Neuron* 84, 292-309. 10.1016/j.neuron.2014.10.019 25374356 PMC4269290

[DMM052029C45] Mann, H. B. and Whitney, D. R. (1947). On a test of whether one of two random variables is stochastically larger than the other. *Ann. Math. Stat.* 18, 50-60. 10.1214/aoms/1177730491

[DMM052029C46] McLaren, W., Pritchard, B., Rios, D., Chen, Y., Flicek, P. and Cunningham, F. (2010). Deriving the consequences of genomic variants with the ensembl api and snp effect predictor. *Bioinformatics* 26, 2069-2070. 10.1093/bioinformatics/btq330 20562413 PMC2916720

[DMM052029C47] Meyer, N. and Rinholm, J. E. (2021). Mitochondria in myelinating oligodendrocytes: slow and out of breath? *Metabolites* 11, 359. 10.3390/metabo11060359 34198810 PMC8226700

[DMM052029C48] Müller, U. K. and Van Leeuwen, J. L. (2006). Undulatory fish swimming: from muscles to flow. *Fish Fish.* 7, 84-103. 10.1111/j.1467-2979.2006.00210.x

[DMM052029C49] Muller, U., Stamhuis, E. and Videler, J. (2000). Hydrodynamics of unsteady fish swimming and the effects of body size: comparing the flow fields of fish larvae and adults. *J. Exp. Biol.* 203, 193-206. 10.1242/jeb.203.2.193 10607529

[DMM052029C50] Murley, A., Sarsam, R. D., Toulmay, A., Yamada, J., Prinz, W. A. and Nunnari, J. (2015). Ltc1 is an er-localized sterol transporter and a component of er–mitochondria and er–vacuole contacts. *J. Cell Biol.* 209, 539-548. 10.1083/jcb.201502033 25987606 PMC4442815

[DMM052029C51] Novarino, G., Fenstermaker, A. G., Zaki, M. S., Hofree, M., Silhavy, J. L., Heiberg, A. D., Abdellateef, M., Rosti, B., Scott, E., Mansour, L. et al. (2014). Exome sequencing links corticospinal motor neuron disease to common neurodegenerative disorders. *Science* 343, 506-511. 10.1126/science.1247363 24482476 PMC4157572

[DMM052029C52] Parichy, D. M. (2015). The natural history of model organisms: Advancing biology through a deeper under- standing of zebrafish ecology and evolution. *Elife* 4, e05635. 10.7554/eLife.05635 25807087 PMC4373672

[DMM052029C53] Parodi, L., Coarelli, G., Stevanin, G., Brice, A. and Durr, A. (2018). Hereditary ataxias and paraparesias: clinical and genetic update. *Curr. Opin. Neurol.* 31, 462-471. 10.1097/WCO.0000000000000585 29847346

[DMM052029C54] Perera, R. P. and Dosch, R. (2021). In vivo imaging of protein interactions in the germplasm with bimolecular fluorescent complementation. *Germline Dev. Zebrafish Method. Protocol.* 2218, 303-317. 10.1007/978-1-0716-0970-5_24 33606241

[DMM052029C55] Saxton, W. M. and Hollenbeck, P. J. (2012). The axonal transport of mitochondria. *J. Cell Sci.* 125, 2095-2104. 22619228 10.1242/jcs.053850 PMC3656622

[DMM052029C56] Schindelin, J., Arganda-Carreras, I., Frise, E., Kaynig, V., Longair, M., Pietzsch, T., Preibisch, S., Rueden, C., Saalfeld, S., Schmid, B. et al. (2012). Fiji: an open-source platform for biological-image analysis. *Nat. Methods* 9, 676-682. 10.1038/nmeth.2019 22743772 PMC3855844

[DMM052029C57] Siems, S. B., Jahn, O., Hoodless, L. J., Jung, R. B., Hesse, D., Möbius, W., Czopka, T. and Werner, H. B. (2021). Proteome profile of myelin in the zebrafish brain. *Front. Cell Dev. Biol.* 9, 640169. 10.3389/fcell.2021.640169 33898427 PMC8060510

[DMM052029C58] Sievers, F., Wilm, A., Dineen, D., Gibson, T. J., Karplus, K., Li, W., Lopez, R., McWilliam, H., Remmert, M., Söding, J. et al. (2011). Fast, scalable generation of high-quality protein multiple sequence alignments using clustal omega. *Mol. Syst. Biol.* 7, 539. 10.1038/msb.2011.75 21988835 PMC3261699

[DMM052029C59] Smits, A. J. (2019). Undulatory and oscillatory swimming. *J. Fluid Mech.* 874, P1. 10.1017/jfm.2019.284

[DMM052029C60] Spence, R. (2011). Zebrafish ecology and behaviour. In *Zebrafish Models in Neurobehavioral Research* (ed. A. Kalueff and J. Cachat), pp. 1-46. Neuromethods, vol. 52. Totowa, NJ: Humana Press. 10.1007/978-1-60761-922-2_1

[DMM052029C61] Tang, Z., Takahashi, Y. and Wang, H.-G. (2019). Atg2 regulation of phagophore expansion at mitochondria- associated er membranes. *Autophagy* 15, 2165-2166. 10.1080/15548627.2019.1666594 31512567 PMC6844525

[DMM052029C62] Tesson, C., Koht, J. and Stevanin, G. (2015). Delving into the complexity of hereditary spastic paraplegias: how unexpected phenotypes and inheritance modes are revolutionizing their nosology. *Hum. Genet.* 134, 511-538. 10.1007/s00439-015-1536-7 25758904 PMC4424374

[DMM052029C63] Wardle, C., Videler, J. and Altringham, J. (1995). Tuning in to fish swimming waves: body form, swimming mode and muscle function. *J. Exp. Biol.* 198, 1629-1636. 10.1242/jeb.198.8.1629 9319534

[DMM052029C64] Weil, M.-T., Möbius, W., Winkler, A., Ruhwedel, T., Wrzos, C., Romanelli, E., Bennett, J. L., Enz, L., Goebels, N., Nave, K.-A. et al. (2016). Loss of myelin basic protein function triggers myelin breakdown in models of demyelinating diseases. *Cell Rep.* 16, 314-322. 10.1016/j.celrep.2016.06.008 27346352 PMC4949381

[DMM052029C65] Weil, M.-T., Ruhwedel, T., Meschkat, M., Sadowski, B. and Möbius, W. (2019). Transmission electron microscopy of oligodendrocytes and myelin. *Oligodendrocytes* 1936, 343-375. 10.1007/978-1-4939-9072-6_20 30820909

[DMM052029C66] Westerfield, M. (2000). The zebrafish book: a guide for the laboratory use of zebrafish (*Danio rerio*), 4th edn. University of Oregon Press. http://zfin.org/zf_info/zf-book/zfbk.html.

[DMM052029C67] Will, U. (1986). Mauthner neurons survive metamorphosis in anurans: A comparative hrp study on the cytoarchitecture of mauthner neurons in amphibians. *J. Comp. Neurol.* 244, 111-120. 10.1002/cne.902440109 3081602

[DMM052029C68] William, S. (1908). The probable error of a mean. *Biometrika* 6, 1-25. 10.2307/2331554

[DMM052029C69] Wilson, E. L. and Metzakopian, E. (2021). Er-mitochondria contact sites in neurodegeneration: genetic screening approaches to investigate novel disease mechanisms. *Cell Death Differ.* 28, 1804-1821. 10.1038/s41418-020-00705-8 33335290 PMC8185109

[DMM052029C70] Yang, J. and Zhang, Y. (2015). I-tasser server: new development for protein structure and function predictions. *Nucleic Acids Res.* 43, W174-W181. 10.1093/nar/gkv342 25883148 PMC4489253

[DMM052029C71] Zhang, C., Freddolino, P. L. and Zhang, Y. (2017). Cofactor: improved protein function prediction by combining structure, sequence and protein–protein interaction information. *Nucleic Acids Res.* 45, W291-W299. 10.1093/nar/gkx366 28472402 PMC5793808

[DMM052029C72] Zhang, Y. and Skolnick, J. (2005). Tm-align: a protein structure alignment algorithm based on the tm-score. *Nucleic Acids Res.* 33, 2302-2309. 10.1093/nar/gki524 15849316 PMC1084323

[DMM052029C73] Zhou, X., Zheng, W., Li, Y., Pearce, R., Zhang, C., Bell, E. W., Zhang, G. and Zhang, Y. (2022). I-TASSER-MTD: a deep-learning-based platform for multi-domain protein structure and function prediction. *Nat. Protoc.* 17, 2326-2353. 10.1038/s41596-022-00728-0 35931779

[DMM052029C74] Zottoli, S. J. and Faber, D. S. (2000). Review: the Mauthner cell: what has it taught us? *Neuroscientist* 6, 26-38. 10.1177/107385840000600111

