# Supplementary material for: Patient-specific mutation of contact site protein Tomm70 causes neurodegeneration
Source: Dis Model Mech. 2025 Apr 28;18(4):dmm052029. doi: 10.1242/dmm.052029 (PMC12067081; doi:10.1242/dmm.052029)
Supplement: Supplementary information [file dmm-18-052029-s1.pdf]

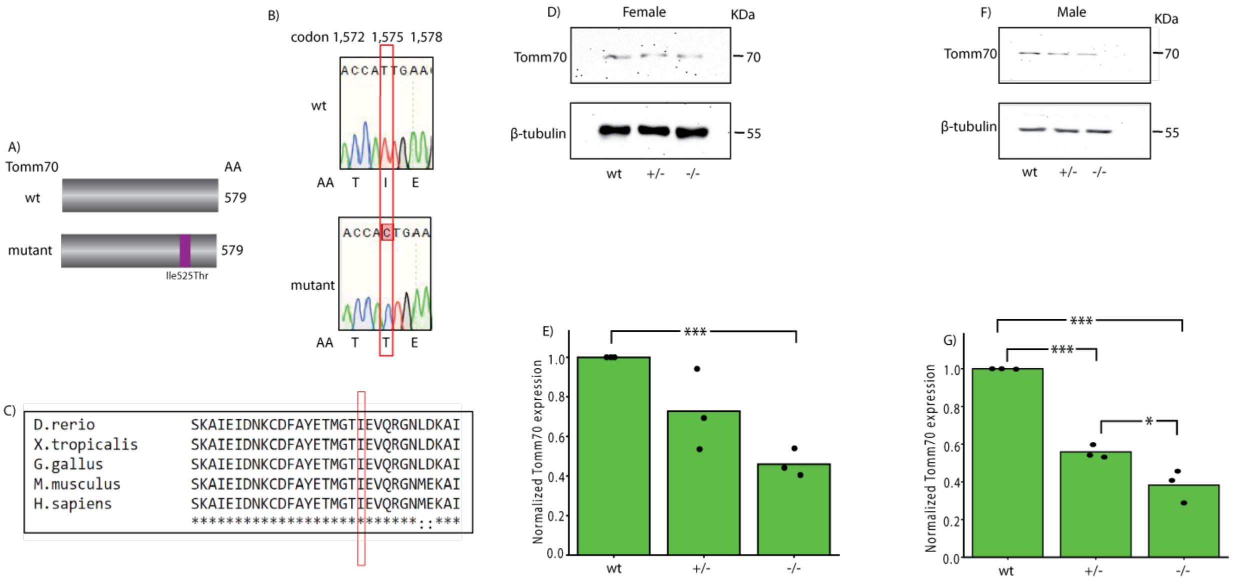

Fig. S1. A single point mutation in the *tomm70* gene and its effects. A) & B) Using RNA sequencing method, it was found that *ruehrei*^{p25ca} fish carry a point mutation in the *tomm70* gene in which a base change from thymine to cytosine leads to the change in an amino acid from isoleucine to threonine at the 525th position- *Danio rerio* Tomm70^{Ile525Thr}. C) Multiple sequence alignment showed that the amino acid isoleucine is highly conserved among vertebrates including humans. D) & F) Immunoblotting of Tomm70 protein in the brain of wild-type and *tomm70* mutant female and male fish, respectively, using anti-β-Tubulin as a control. E) & G) Bar graph shows relative abundance of Tomm70 among the three genotypes in female and male fish, respectively. There is a significant decrease in the abundance of Tomm70 in the brain of mutant fish compared to the wild types. Data is represented as median and dots represent individual data points. (n, no. of experimental repeats = 3). Statistical significance was calculated with independent t test. ***$p < 0.001$.

Disease Models & Mechanisms • Supplementary information

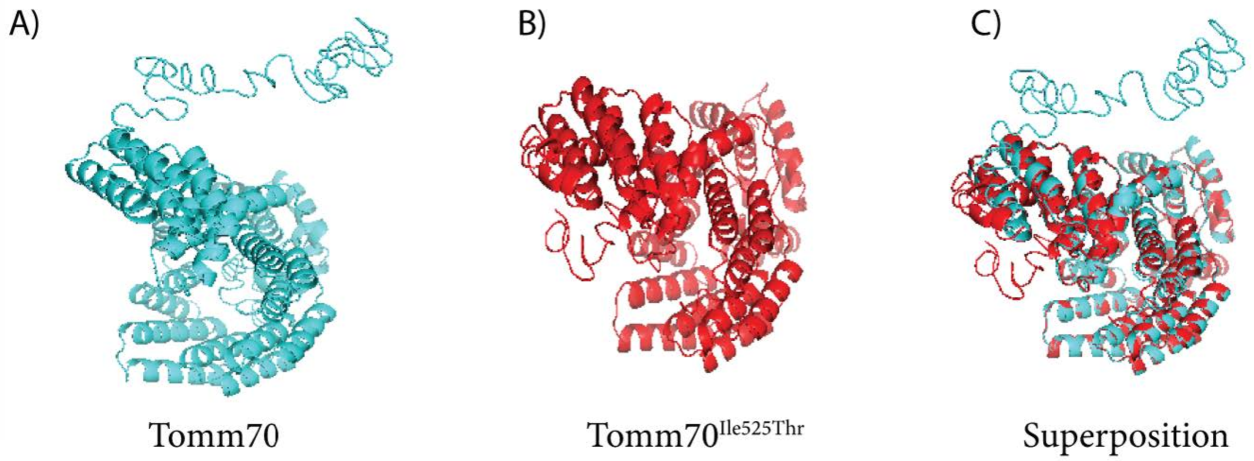

Fig. S2. No change in the structural conformation of Tomm70 due to the mutation. The homology models for wild-type and mutant Tomm70 were predicted by the I-TASSER. Superposition of wild-type and mutant Tomm70 structures was done using PyMol and TM-align software of I-TASSER. A) The homology model with highest C-score (-0.26) for wild-type Tomm70. B) The homology model with highest C-score (-0.28) for mutant Tomm70^{Ile525Thr}. C) Superposition of models for wild-type and mutant Tomm70. The root-mean-square deviation (RMSD) = 0.233 and TM align score = 0.83586. The RMSD value below 2 Å and a TM score above 0.5 indicates that the two structures are nearly identical.

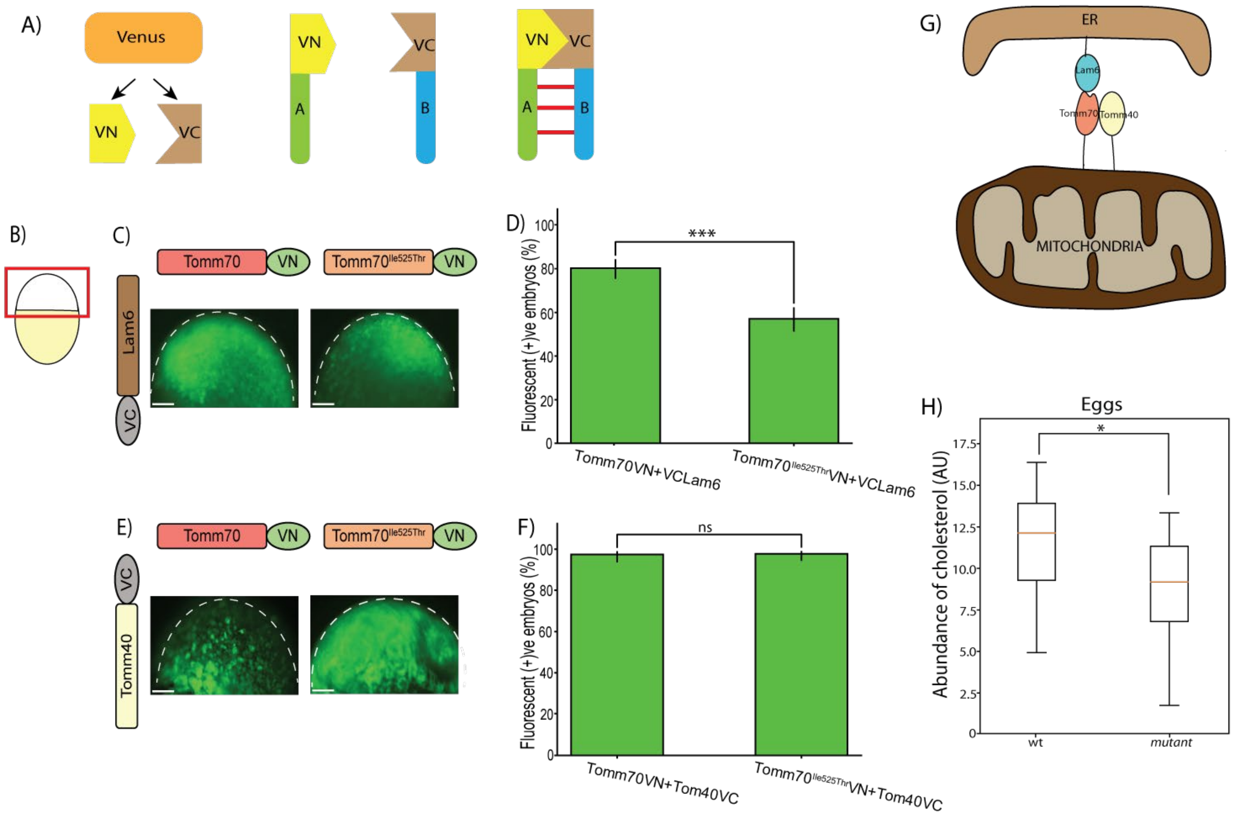

Fig. S3. Effect of mutation on the interaction of Tomm70 with its partners. A)The cartoon illustrate the working of BiFC assay. The candidate proteins A and B are tagged with the split parts of the ”Venus” fluorescent reporter protein ”VN” and ”VC”, respectively. The interaction of two proteins brings the split part in close proximity allowing reporter protein to regain its native conformation and start to fluoresce. B) The red box in the cartoon represents the animal pole side of the zebrafish embryo which is the region imaged in further pictures. C) & E) Representative pictures of zebrafish embryos, 7-8 h post fertilisation showing a fluorescent signal and confirming the interaction of Lam6 and Tomm40 with wild-type and mutant Tomm70. (Scale bar: 100 μm). D) Quantification of the percentage of fluorescent positive embryos with injection of Tomm70-VN+VC-Lam6 and Tomm70^{Ile525Thr}-VN+VC-Lam6 constructs. The data is a representation of 6 independent experiments. (n, total no of embryos counted for Tomm70-VN+VC-Lam6 and Tomm70^{Ile525Thr}-VN+VC-Lam6 injection: 301 and 303, respectively). F) Quantification of the percentage of fluorescent positive embryos with injection of Tomm70-VN+Tomm40-VC and Tomm70^{Ile525Thr}-VN+Tomm40- VC constructs. The data is a representation of 3 independent experiments. (n, total no of embryos counted for Tomm70-VN+Tomm40-VC and Tomm70^{Ile525Thr}-VN+Tomm40-VC construct: 157 and 176, respectively). Error bar represents 95% confidence interval. G) The current model showing the effect of the mutation on the interaction of Tomm70 with Lam6 at the ER-mitochondria contact site. H) Data is represented as box plot for relative abundance of cholesterol in the wild-type and opaque eggs, normalized to the internal standard and mass of the sample. The orange line shows the median, the box displays the upper and lower quartile, the whiskers denote 1.5 times the interquartile range. (N, no. of fish from which eggs collected, 20 for wild-type and 10 for mutant. n, no. of eggs from each fish was approximately 50). Statistical significance was tested using Fisher ’s permutation test for the injection data and with Wilcoxon rank-sum test for cholesterol data. *$p < 0.05$ and ***$p < 0.001$ and ns is non-significant.

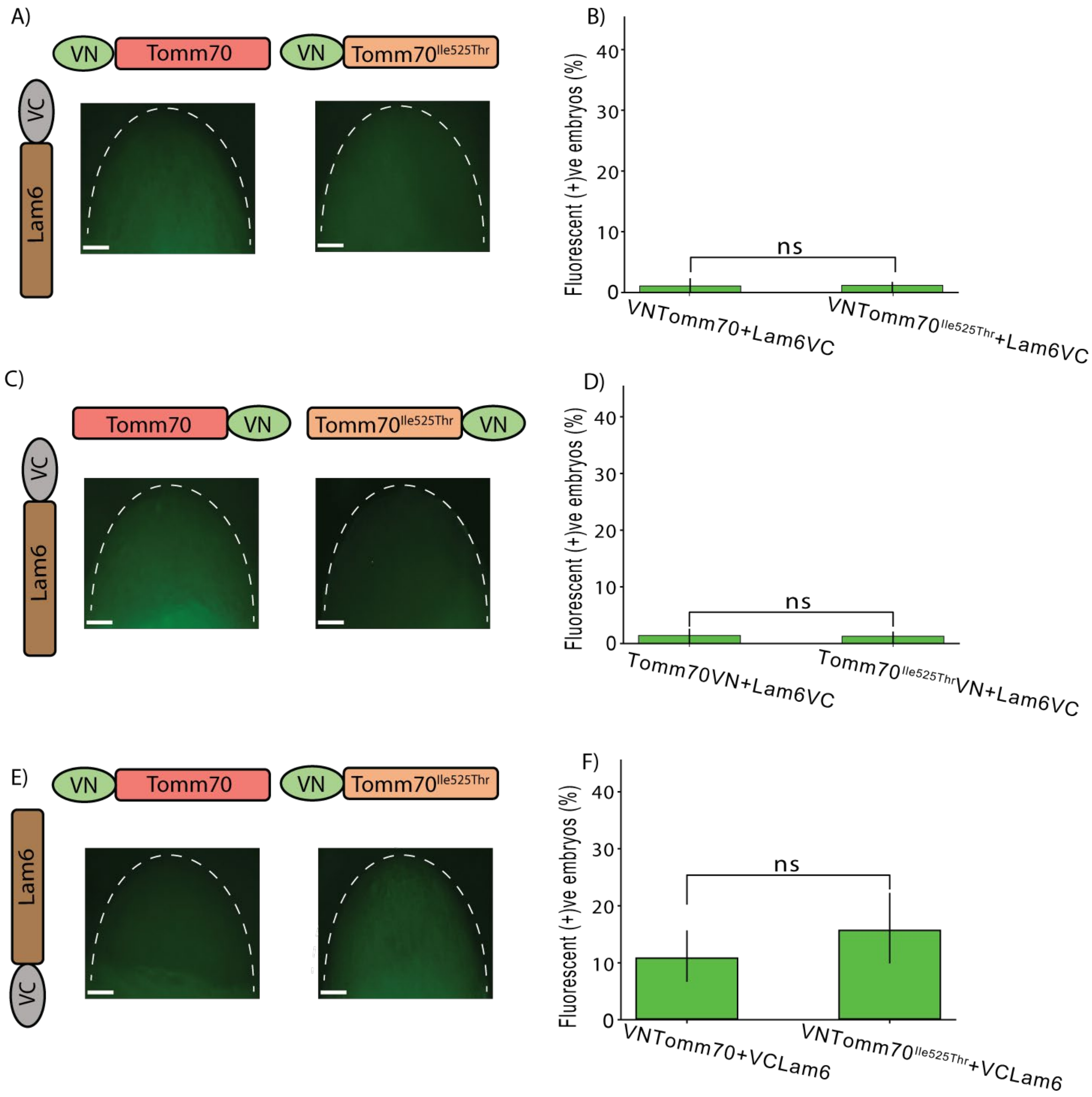

Fig. S4. **BiFC control combinations for the interaction of Tomm70 and Lam6.** A) & C) Representative pictures of zebrafish embryos, 7-8 h post fertilisation showing no fluorescent signal for the combination of Lam6-VC with wild-type and mutant Tomm70 with VN attached to its N and C-terminus, respectively. (Scale bar: 100 μm).

Disease Models & Mechanisms • Supplementary information

B) Quantification of the percentage of fluorescent positive embryos for the injection of VN-Tomm70+Lam6-VC and VN-Tomm70^{Ile525Thr}+Lam6-VC constructs. The data is a representation of 3 independent experiments. (n, total no of embryos counted for VN-Tomm70+Lam6-VC and VN-Tomm70^{Ile525Thr}+Lam6-VC injection: 156 and 152, respectively). D) Quantification of the percentage of fluorescent positive embryos for the injection of Tomm70-VN+Lam6-VC and Tomm70^{Ile525Thr}-VN+Lam6-VC constructs. The data is a representation of 3 independent experiments. (n, total no of embryos counted for Tomm70-VN+Lam6-VC and Tomm70^{Ile525Thr}- VN+Lam6-VC injection: 138 and 168, respectively). E) Representative images of zebrafish embryos, 7-8 h post fertilisation showing no fluorescent signal for the combination of VC-Lam6 with VN-Tomm70 and VN-Tomm70^{Ile525Thr}. (Scale bar: 100 μm). F) Quantification of the percentage of fluorescent positive embryos for the injection of VN-Tomm70+VC-Lam6 and VN-Tomm70^{Ile525Thr}+VC-Lam6 constructs. Only about 10-15% embryos possessed a very light fluorescent signal intensity for both the combinations. The data is a representation of 3 independent experiments. (n, total no of embryos counted for VN-Tomm70+VC-Lam6 and VN-Tomm70^{Ile525Thr}+VC-Lam6 injection: 177 and 128, respectively). Error bar represents 95% confidence interval. Statistical significance was tested using Fisher's permutation test. ns is non-significant.

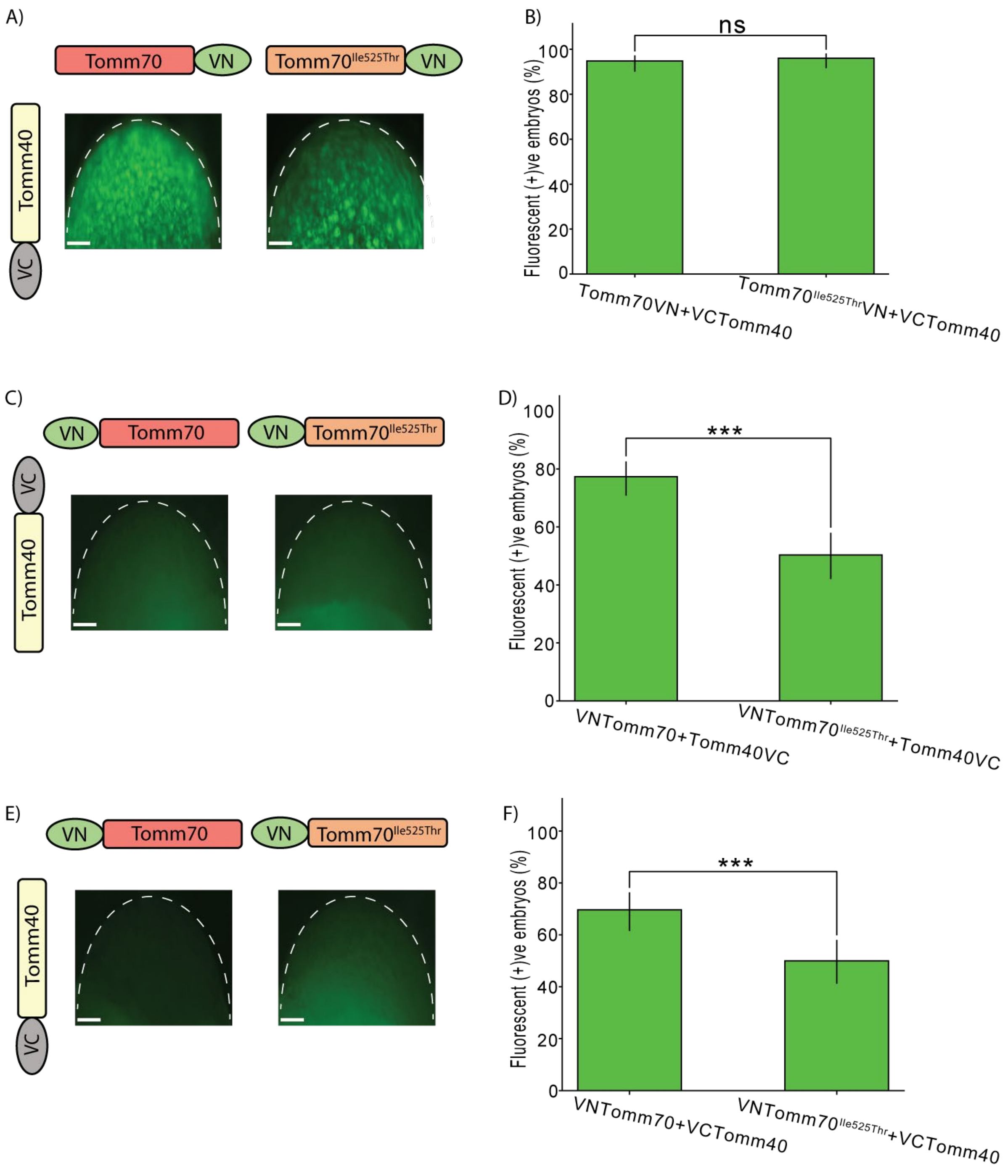

Disease Models & Mechanisms • Supplementary information

Fig. S5. BiFC control combinations for the interaction of Tomm70 and Tomm40. A) Representative pictures of zebrafish embryos, 7-8 h post fertilisation showing a clear fluorescent signal for the combination of VC-Tomm40 with Tomm70-VN and Tomm70^{Ile525Thr}-VN. (Scale bar 100 µm). B) Quantification of the percentage of fluorescent positive embryos for the injection of Tomm70-VN+VC-Tomm40 and Tomm70^{Ile525Thr}- VN+VC-Tomm40 constructs. There is no change in the percentage of fluorescent positive embryos for the interaction of wild-type and mutant Tomm70 with Tomm40 in this combination. The data is a represen- tation of 3 independent experiments. (n, total no of embryos counted for Tomm70-VN+VC-Tomm40 and Tomm70^{Ile525Thr}-VN+VC-Tomm40 injection: 155 and 153, respectively) C) & E) Representative pictures of zebrafish embryos, 7-8 h post fertilisation showing no clear fluorescence signal for the combination of VN-Tomm70 and VN-Tomm70^{Ile525Thr} with Tomm40 when VC is attached to its C and N-terminus, re- spectively. (Scale bar: 100 µm). D) Quantification of the percentage of fluorescent positive embryos for the injection of VN-Tomm70 +Tomm40-VC and VN-Tomm70^{Ile525Thr}+Tomm40-VC constructs. Although there was a significant difference in the percentage of fluorescent positive embryos, this is of least biological significance as fluorescence signal intensity was very low in this combination. The data is a representation of 3 independent experiments. (n, total no of embryos counted for VN-Tomm70+Tomm40-VC and VN-Tomm70^{Ile525Thr}+Tomm40-VC injection: 194 and 149, respectively). F) Quantification of the percentage of fluorescent positive embryos for the injection of VN-Tomm70+VC-Tomm40 and VN-Tomm70^{Ile525Thr} +VC- Tomm40 constructs. Similar results were obtained for this combination with a significant difference in the percentage of fluorescent positive embryos which possess very weak signal intensity. The data is a representation of 3 independent experiments. (n, total no of embryos counted for VN-Tomm70+VC-Tomm40 and VN-Tomm70^{Ile525Thr}+VC-Tomm40 injection: 145 and 132, respectively). Error bar represents 95% confidence interval. Statistical significance was tested using Fisher's permutation test. ns is non-significant.

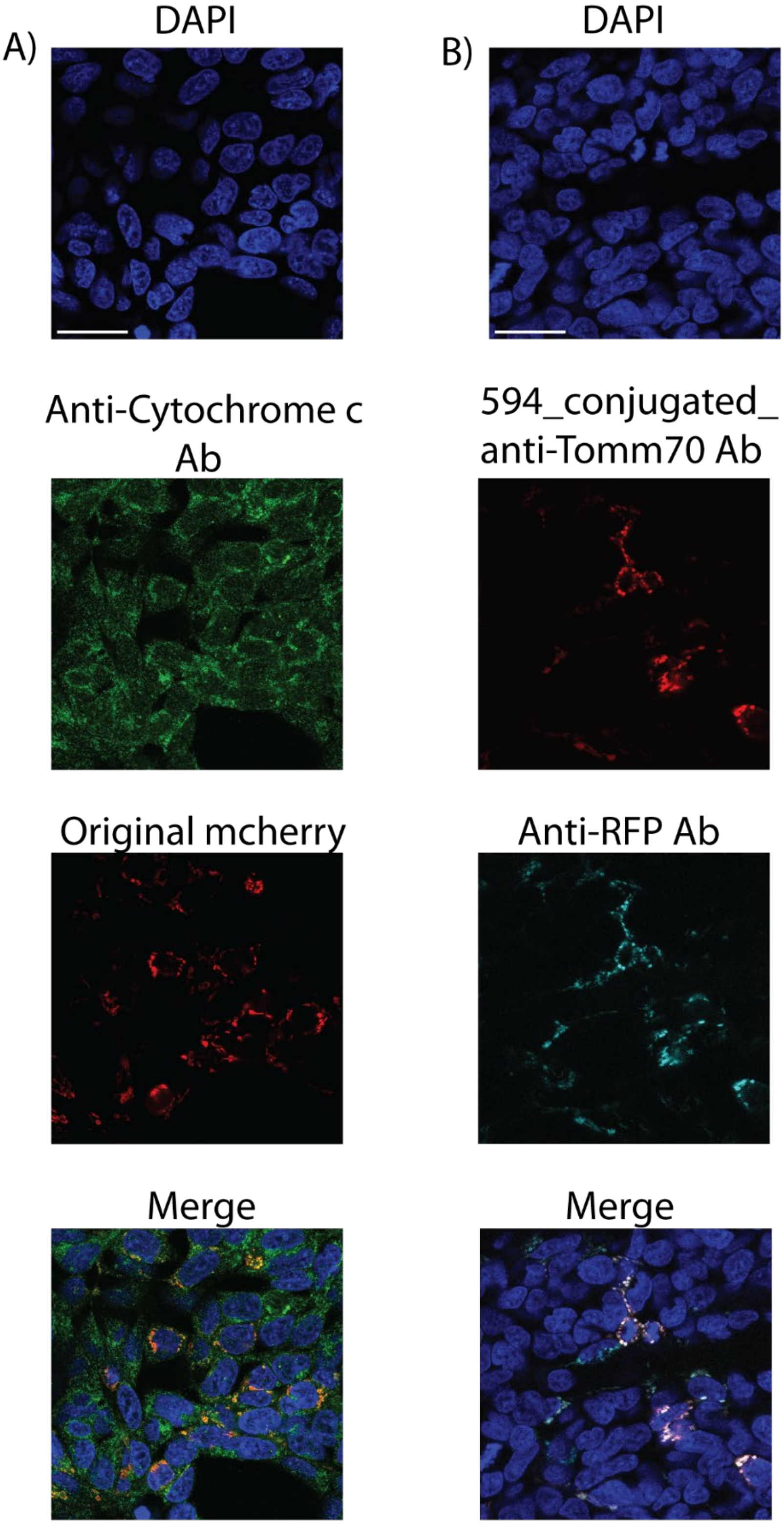
A)
DAPI
Anti-Cytochrome c Ab
Original mcherry
Merge
B)
DAPI
594_conjugated_ anti-Tomm70 Ab
Anti-RFP Ab
Merge

Fig. S6. Antibody specificity confirmation using HEK cells. The brightness of images was corrected using Image J. A) HEK cells expressing mCherry-Mito-7 were stained with DAPI and anti-Cytochrome c antibody. A similar pattern and colocalization of the Cytochrome c signal with original mCherry confirms the specificity of the anti-Cytochrome c antibody.

B) HEK cells expressing mCherry-Mito-7 plasmid were stained with DAPI, 594-conjugated-anti-Tomm70, and anti-RFP antibody (for staining mCherry) after killing the original m-cherry signal with an increased amount of 4% PFA. A similar pattern and co-localization of Tomm70 and RFP confirms the specificity of the 594-conjugated-anti-Tomm70 antibody. (Scale bar: 30 μm).

Disease Models & Mechanisms • Supplementary information

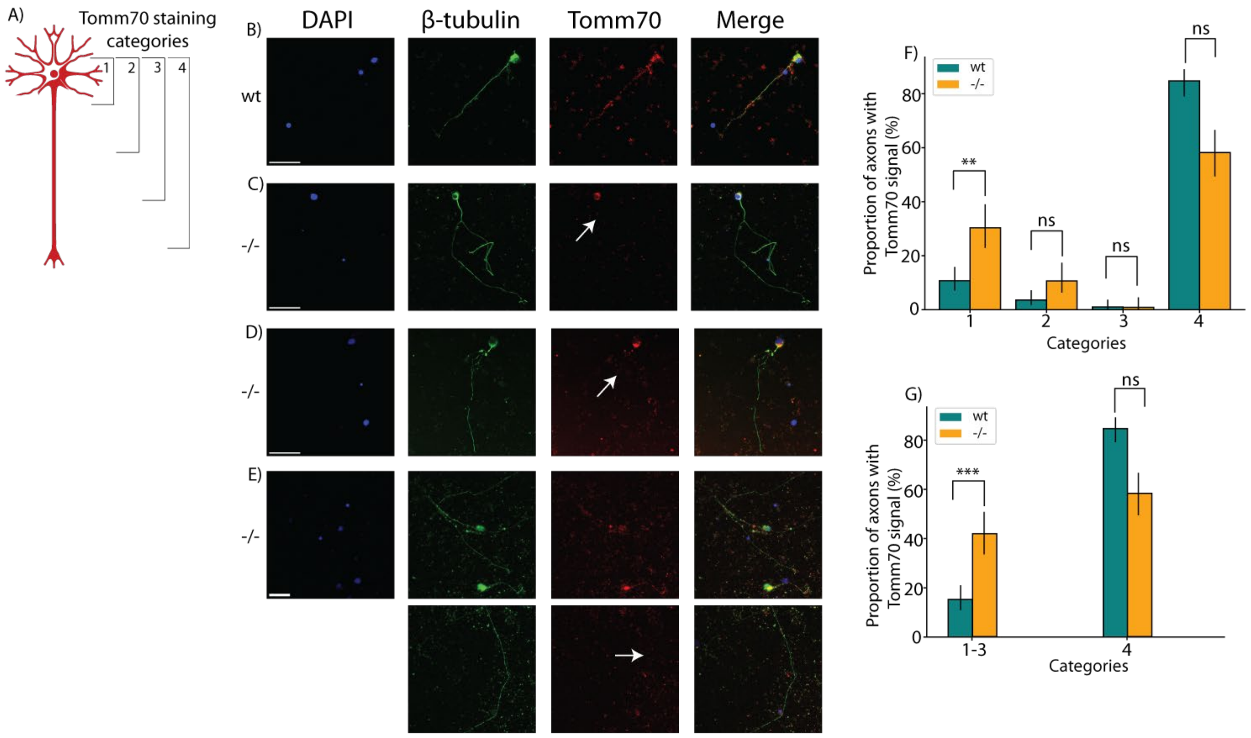

Fig. S7. Absence of Tomm70 from the axons in a length dependent manner. The cultured brain neurons of wild-type and *tomm70* mutant male fish were stained with DAPI (representing nucleus) (blue), anti-β-Tubulin antibody (a neuronal marker) (green) and anti-Tomm70 antibody (red). The brightness of images was corrected using Image J. A) The cartoon represents four different categories of staining observed during imaging and quantification of the wild-type and *tomm70* mutant male brain cultured neurons stained with anti-Tomm70 antibody. Category 1, 2, 3, and 4 depict the location of the signal for Tomm70 in the neurons.
B) Representative picture of a neuronal staining in the category 4 from the wild-type male fish possessing signal for Tomm70 both in the soma and axon. C) Representative picture of a neuronal staining in the category 1 from the mutant male fish having signal for Tomm70 only in the soma. D) Representative picture of a neuronal staining in the category 2 from the mutant male fish having signal for Tomm70 in the soma and in the initial part of the axon. E) Representative picture of a neuronal staining in the category 3 from the mutant male fish having signal for Tomm70 in the soma and half way to the axon but not in its full length. The white arrows in the representative pictures marks the absence of Tomm70 signal in the axon. (Scale bar: 30 µm). F) Quantification representing the percentage of neuronal staining in 4 different categories for the wild types and mutants. More than 80% of the neuronal staining in the wild types belong to the category
4. On the other hand, there is a significant increase in the percentage of neuronal staining in category 1 in the mutants. Although not significant, but there is still an increase in neuronal staining in category 2 and 3 and a decrease in category 4 in the mutants. G) Quantification of the percentage of neuronal staining in category 1-3 together and for category 4 in wild-type and mutant fish. There is a significant increase in the percentage of neuronal staining in category 1-3 in mutants compared to the wild types.(N, no of fish wt = 9 and -/- = 7. n, total number of neurons counted wt = 196 , -/- = 122). Error bar represents 95% confidence interval. Statistical significance was tested using Fisher's permutation test. $**p < 0.01$, $***p < 0.001$ and ns is non-significant.

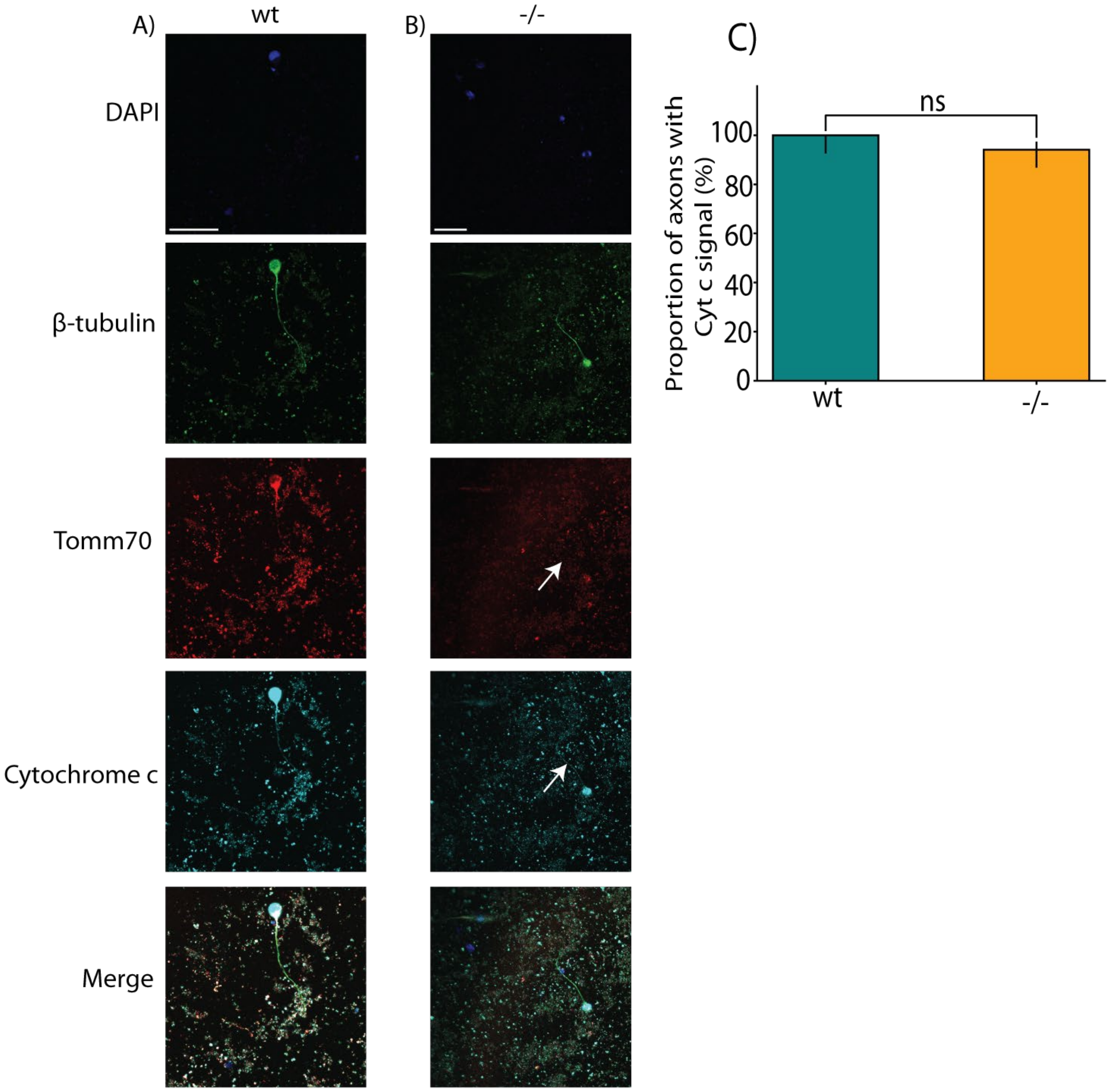

Fig. S8. Mutation influences the mitochondrial transport to the axons. The cultured brain neurons of wild-type and *tomm70* mutant male fish were stained with DAPI (representing nucleus) (blue), anti-*β*-Tubulin antibody (a neuronal marker) (green) and anti-Tomm70 antibody (red) and anti-Cytochrome c antibody (a conserved mitochondrial marker) (cyan). The brightness of images was corrected using Image J. A) Representative picture of a neuronal staining from wild-type male fish showing a signal for Tomm70 and Cytochrome c in the axon. B) Representative picture of a neuronal staining from mutant male fish, with white arrows showing no signal for Tomm70 but for Cytochrome c in the axon. (Scale bar: 30µm). C) Quantification of the percentage of neuronal staining showing a signal for Cytochrome c in the axons in wild-type and *tomm70* mutant male. Quantifications for Tomm70 signals are given in Figure 7G. (N, no of fish wt = 4 and -/- = 4. n, total number of neurons counted wt = 48, -/- = 85). Error bar represents 95% confidence interval. Statistical significance was tested using Fisher's permutation test. ns is non-significant

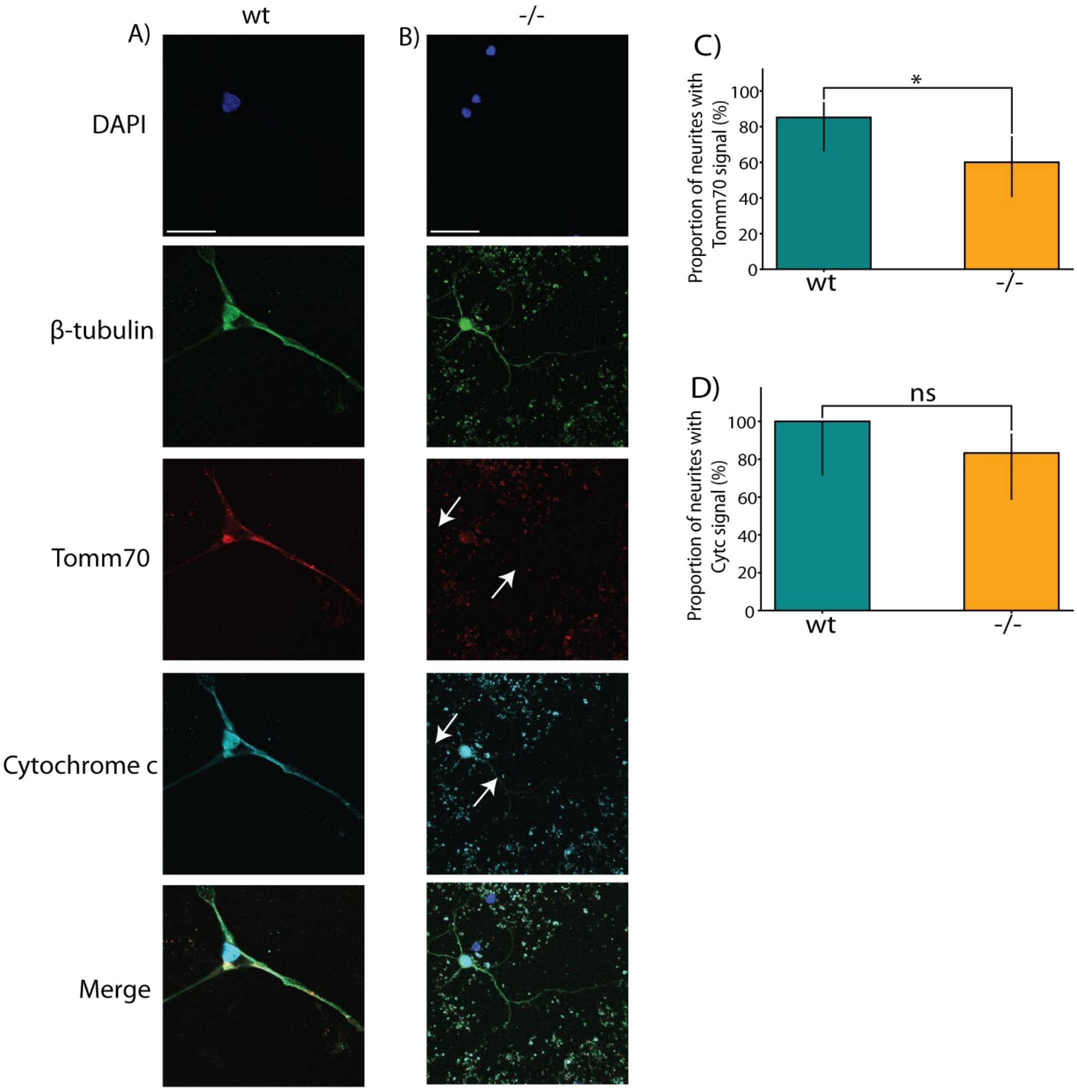

Fig. S9. **Mutation impacts the transport of mitochondria to the dendrites as well.** The cultured brain neurons of wild-type and *tomm70* mutant male fish were stained with DAPI (representing nucleus) (blue), anti-β-Tubulin antibody (a neuronal marker) (green), anti-Tomm70 antibody (red), and anti-Cytochrome c antibody (a conserved mitochondrial marker) (cyan). The brightness of images was corrected using Image J. A) Representative picture of a multi-polar neuronal staining from wild-type male fish showing a signal for Tomm70 and Cytochrome c in all the neurites. B) Representative picture of a multi-polar neuronal staining from mutant male fish, with white arrows showing no signal for Tomm70 but for Cytochrome c in the neurites. (Scale bar: 30 μm). C) Quantification of the percentage of neuronal staining showing a signal for Tomm70 in the neurites in wild-type and mutant male fish. D) Quantification of the percentage of neuronal staining showing a signal for Cytochrome c in the neurites in wild-type and mutant male fish. (N, no of fish wt = 9 and -/- = 7 for Tomm70 and no of fish wt = 4 and -/- = 4 for Cytochrome c. n, total number of multi-polar neurons counted wt = 27, -/- = 30 for Tomm70 and wt = 11, -/- = 18 for Cytochrome c signal). Error bar represents 95% confidence interval. Statistical significance was tested using Fisher's permutation test. *p <0.05 and ns is non-significant.

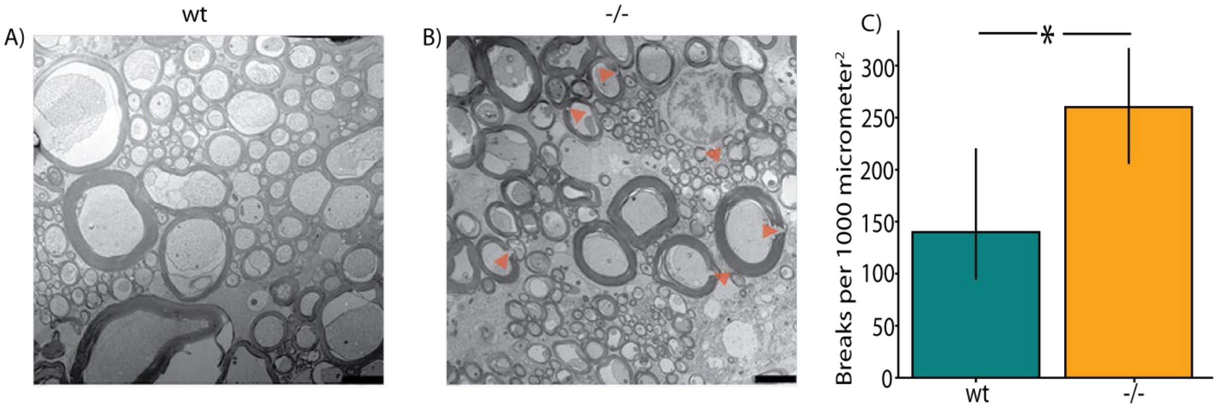

Fig. S10. Myelin phenotype in the large calibre axons of the spinal cord of *tomm70* mutants. A) Representative electron microscopy picture showing large calibre axons in the caudal part of the spinal cord with intact myelin from wild-type fish (Scale bar: 2500 nm). B) Arrow heads mark breaks of the myelin sheath in the large calibre axons of the caudal part of the spinal cord from homozygous mutants (Scale bar: 2500 nm). C) Quantification for the number of breaks of the myelin sheath analysed per 1000 micrometer^2 area between wild-type and mutant fish. There is a significant increase in the number of breaks of myelin sheath in mutants compared to the wild types in the caudal part of the spinal cord. (N, no. of fish wt = 3, -/- = 6). Error bar represents 95% confidence interval. Statistical significance was tested using Fisher's permutation test. *$p < 0.05$

Disease Models & Mechanisms • Supplementary information

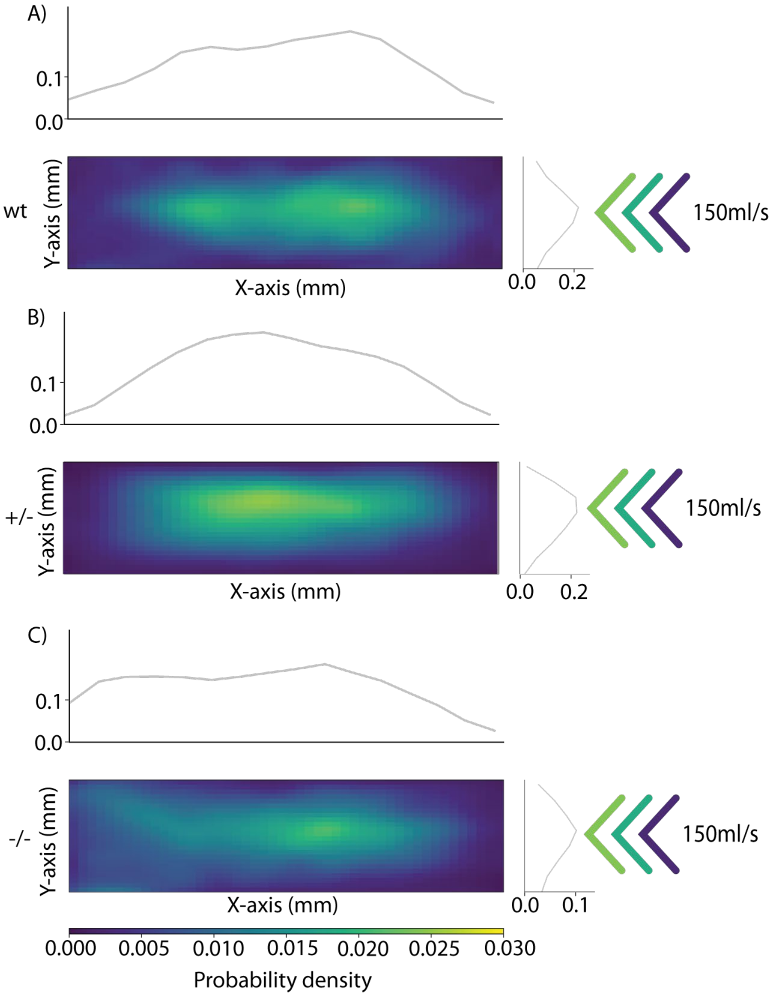

Fig. S11. *tomm70* mutant male fish avoid the centre of the stream. The 2-D heat map with marginal histogram showing all possible locations of male fish in the set up. The blue colour represents the low location probability and yellow represents high location probability. A), B), & C) While the wild-type and heterozygous mutant male fish remain in the centre of the stream, the homozygous mutants avoid it and reside at a longer distance from the centre of the stream. (N, no. of fish, male (wt = 46, +/- = 60, -/- = 75).

Disease Models & Mechanisms • Supplementary information

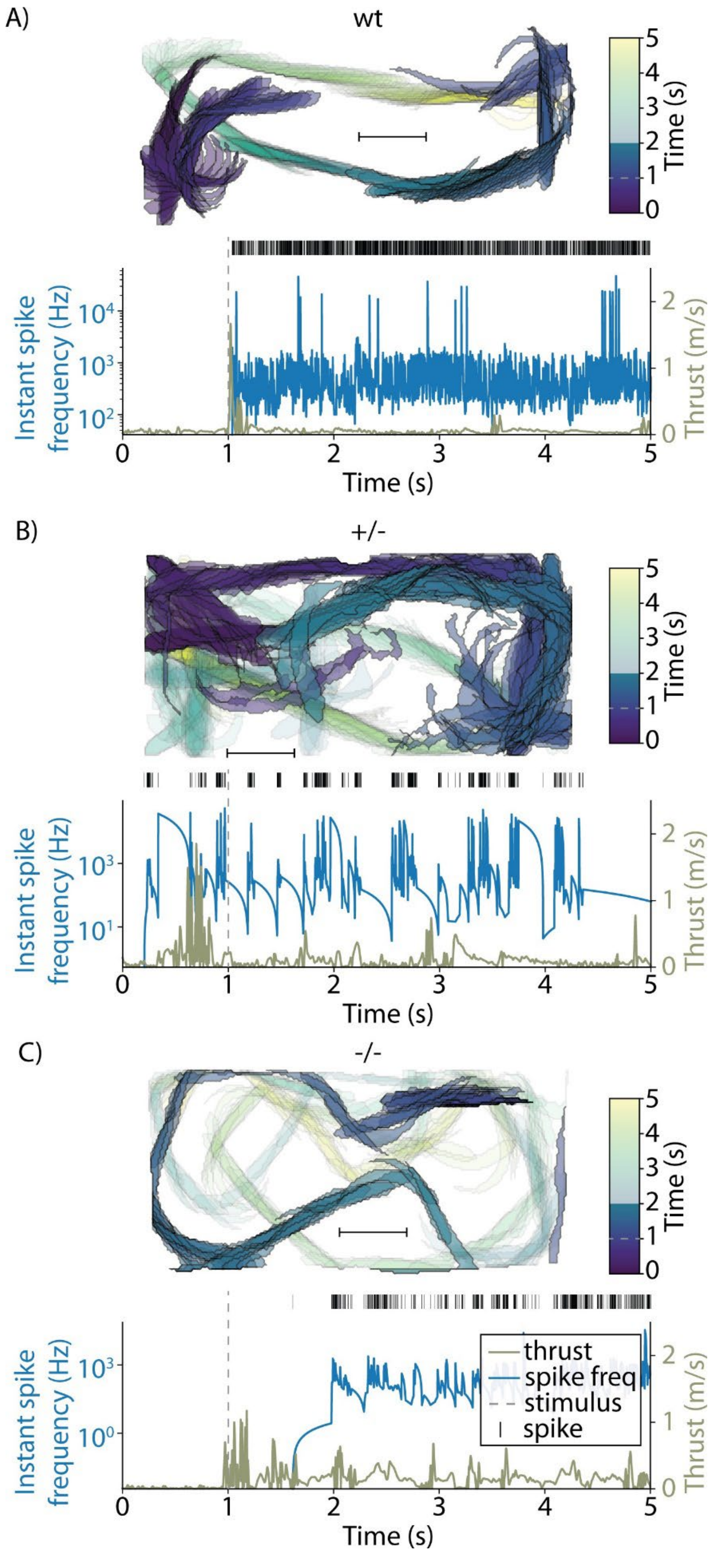
A)
wt
Time (s)
Instant spike frequency (Hz)
Thrust (m/s)
Time (s)
B)
+/-
C)
-/-
thrust
spike freq
stimulus
spike

Fig. S12. Impact of Tomm70^{Ile525Thr} mutation on M-cell mediated C-start escape response in male zebrafish. A-C) display trajectory traces and corresponding kinematic data of male wild-type, heterozygous, and homozygous mutant zebrafish during electrophysiological recordings. Sequential outlines of the zebrafish motion are captured at 25 ms intervals, colour-coded according to the colour bar, which also indicates the stimulus delivery period with a grey dashed line. For enhanced visibility of the pivotal response phase, the first two seconds have increased opacity. The scale bar corresponds to 1 mm. Below the trajectories, large neural activity spikes are visualised in a raster plot, while the thrust magnitude and instantaneous spike frequency are plotted below as a line graph. Immediate spike response is evident in wild-type specimens upon stimulus presentation, whereas a noticeable response delay is observed in heterozygous mutants, with an even greater delay in homozygous mutants. The grey dashed line consistently indicates the time of stimulus onset. N, no. of fish, male (wt = 42, +/- = 56, and -/- = 74).

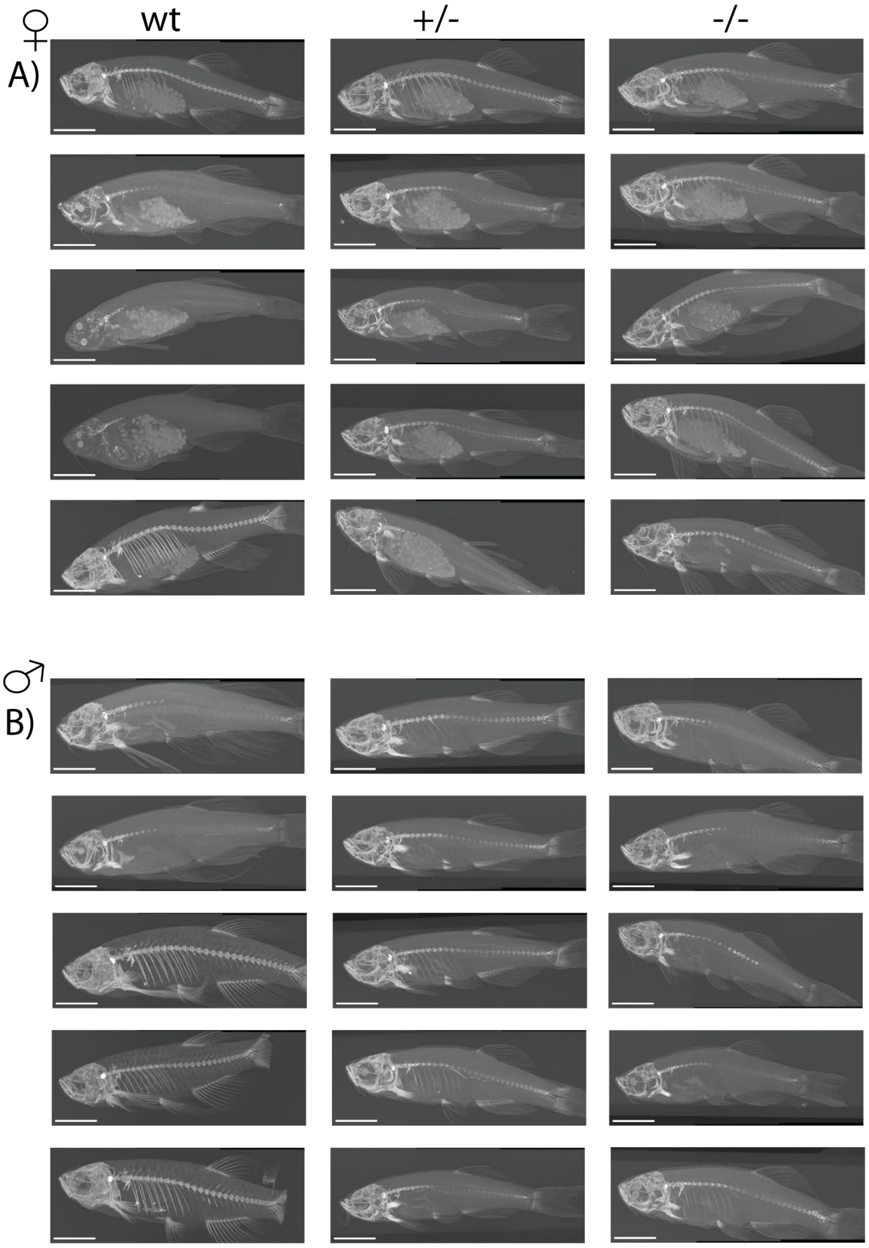

Fig. S13. No skeletal changes in *tomm70* mutants. A) & B) Computed tomography images of the vertebral column of wild type, heterozygous, and homozygous *tomm70* mutant female and male fish. There is no change in the skeletal structure among the three genotypes in both sex. (Scale bar: 5 mm). (N, no. of fish analysed, female, wt = 5, +/- = 5, -/- = 5 and male, wt = 5, +/- = 5, -/- = 5).

Disease Models & Mechanisms • Supplementary information

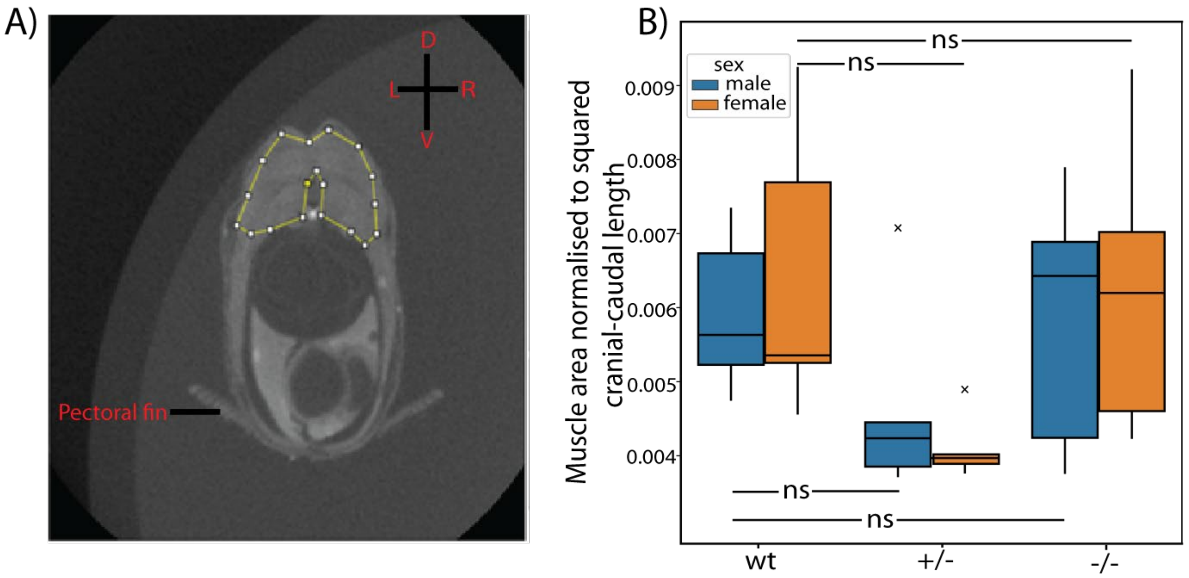

Fig. S14. No muscle atrophy in *tomm70* mutants. A) Exemplary illustration of the fish body in transverse plane with the caudal muscles marked with the yellow segmented line. (D: Dorsal axis, V: Ventral axis, L: Left axis, R: Right axis). B) Box plot of caudal muscle area normalised to squared length of the fish body. The black line represents the median of all individuals, the box displays the upper and lower quartile, the whiskers denote 1.5 times the interquartile distance, and x represent outliers. There is a slight decrease in the muscle area of both heterozygous male and female fish compared to the wild-type and homozygous male and female, respectively. But there is no change in the muscle area between wild-type and homozygous male and female. (N, no. of fish analysed, female, wt = 5, +/- = 5, -/- = 5 and male, wt = 5, +/- = 5, -/- = 5) Statistical significance was tested using Fisher's permutation test, ns is non-significant.

Disease Models & Mechanisms • Supplementary information
